# Impact of conformation and intramolecular interactions on vibrational circular dichroism spectra identified with machine learning

**DOI:** 10.1038/s42004-023-00944-z

**Published:** 2023-07-12

**Authors:** Tom Vermeyen, Ana Cunha, Patrick Bultinck, Wouter Herrebout

**Affiliations:** 1 Department of Chemistry, University of Antwerp, Groenenborgerlaan 171, Antwerpen, 2020 Belgium; 2 Department of Chemistry, Ghent University, Krijgslaan 281, Gent, 9000 Belgium

**Keywords:** Cheminformatics, Circular dichroism, Infrared spectroscopy, Computational chemistry

## Abstract

Vibrational Circular Dichroism (VCD) spectra often differ strongly from one conformer to another, even within the same absolute configuration of a molecule. Simulated molecular VCD spectra typically require expensive quantum chemical calculations for all conformers to generate a Boltzmann averaged total spectrum. This paper reports whether machine learning (ML) can partly replace these quantum chemical calculations by capturing the intricate connection between a conformer geometry and its VCD spectrum. Three hypotheses concerning the added value of ML are tested. First, it is shown that for a single stereoisomer, ML can predict the VCD spectrum of a conformer from solely the conformer geometry. Second, it is found that the ML approach results in important time savings. Third, the ML model produced is unfortunately hardly transferable from one stereoisomer to another.

## Abstract (web summary)

Vibrational circular dichroism is a spectroscopic technique used to study conformational properties and determine absolute configurations of molecules, but spectra are laborious to interpret. Here, the authors use machine learning to assign absolute configurations and to predict conformer spectra solely from molecular geometries.

## Introduction

Chiroptical spectroscopic methods measure the difference in interaction between an optically active compound and left- or right-circularly polarized radiation^1–4^. The best known chiroptical method is Electronic Circular Dichroism (ECD), where one measures the difference in absorption of left- and right-handed circularly polarized visible and ultraviolet radiation by an optically active molecule. Vibrational Circular Dichroism (VCD) is an infrared chiroptical method where vibrational transitions are responsible for the difference in absorption. The main advantage of VCD compared to ECD is the richer information obtained from the former due to the much larger number of vibrational transitions compared to the number of accessible electronic transitions. Chiroptical methods find their main area of application in establishing the absolute configuration (AC) of molecules^4–25^. However, it also reveals a significant amount of information on the conformational properties of a molecule^26–39^. The link between conformation in the sense of its molecular geometry and its VCD spectrum is not easily established on the basis of e.g., some rules of thumb and one usually relies on the quantum chemically computed spectrum. The usual approach to establishing the AC of a compound is to choose a specific AC of the molecule, find all its conformers on the potential energy hypersurface and their energies and then combine all computed spectra using Boltzmann weighting in a simulated molecular spectrum for the chosen AC^1^. By repeating all these steps for each possible AC and comparing all computed spectra to the experimental one, one concludes what AC the experimental sample corresponded to. Said computed spectra are usually generated using Density Functional Theory (DFT) calculations requiring sufficient expertize and computational resources. Experience shows that the VCD spectra of individual conformers of the same molecule may differ significantly even if they belong to the same AC (see Supplementary Discussion 1 and Supplementary Figs. 1–3), explaining why rules of thumb cannot be established^26–34^. The first hypothesis tested in this paper is that machine learning (ML) can be used to predict the VCD spectrum for a specific conformer using only its geometry, in this sense providing an alternative to the DFT procedure. The second hypothesis of this paper is that ML may help reduce significantly the total time cost needed to obtain a molecular spectrum. This entails that ML should allow skipping enough time normally spent in DFT calculations to more than compensate for the time it takes to establish the ML model. The third and final issue examined is the extent to which an ML model is transferable from one AC to another. Does it suffice to learn from one AC and use this for all other possible AC’s? For instance, in a molecule with two stereocenters, does it suffice to establish an ML model for RR and to then use it also for RS, SR, and SS?

**Fig. 1:**
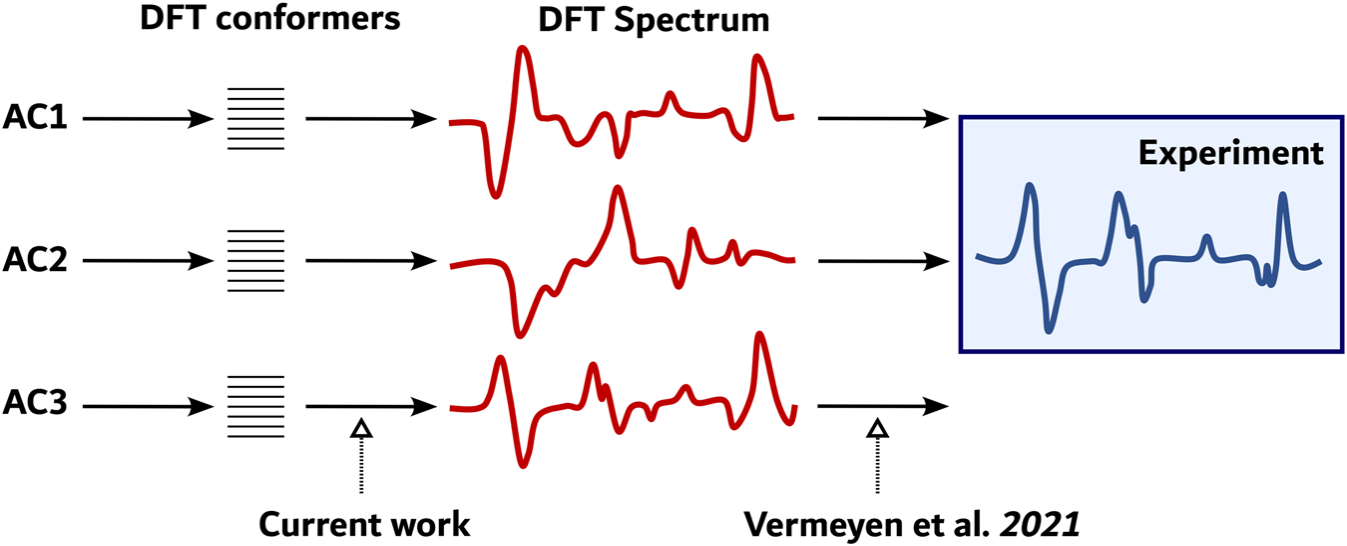
Role of machine learning in VCD. Scope of the current paper: can ML extract the link between the structure of a conformer for a specific AC of a compound and its corresponding DFT computed VCD spectrum? Note the difference with our previous ML work^52^.

ML methods are powerful methods for the extraction of complex patterns hidden in spectral data, speeding up conventional workflows, and accelerating computational methods^40–51^. We have recently shown that there is hope that ML can play a role in VCD spectroscopy^52^. More specifically, we have shown that for a large set of congeneric molecules adopting a single conformer, we can use ML to reveal to what AC a VCD spectrum of an unknown molecule corresponds. Now the ambitions are higher. We want to extract a VCD spectrum solely from a conformer geometry. Figure 1 contrasts the current work against our previous work^52^ and other recent works^53–55^ that address the link between an AC and an experimental spectrum or property. The present paper concentrates on the link between the structure of a conformer and its VCD spectrum within a given AC of a molecule. Success in establishing this link will then also strongly benefit the usual approach to VCD-based AC assignments as it will allow circumpassing the quantum chemical calculation of all conformer VCD spectra.

To prove or disprove the hypotheses set, a test bench of compounds must be established. Somewhat naively, one could think of any set of compounds for which VCD has been computed and/or measured but this is not useful. We namely wish to be able to control ourselves the degree of conformational flexibility of the molecules and the nature of their intramolecular interactions by changing a number of substituents. At the same time, both effects should not intercorrelate too much. This entails the use of admittedly somewhat peculiar molecules but the priority is given to stepwise understand and prove the hypotheses. This would not be possible using too diverse compounds while at the same time, error cancellation could play a much larger role there. We use a tetra-substituted naphthalene framework whose conformational flexibility and intramolecular interactions (such as hydrogen bonding) we can control by judicious selection of substituents. This allows us to test whether ML is sufficiently reliable over a range of chemical situations.

**Fig. 2:**
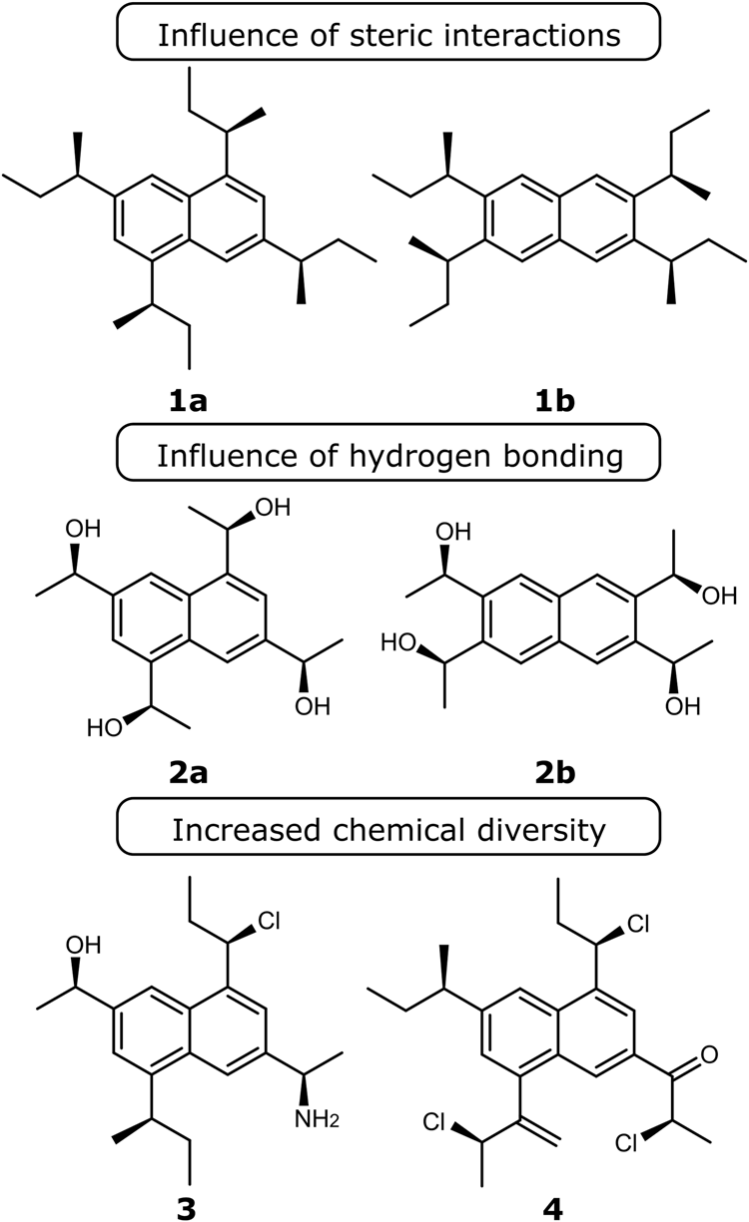
Test compounds for VCD machine learning. Overview of compounds for which the link between conformer and VCD spectrum is extracted with the ML workflow.

To be able to impact the conformational flexibility and the degree to which intramolecular interactions play a role without changing too many features simultaneously, we have chosen compounds that have the same backbone. A tetra-substituted naphthalene framework is chosen as backbone. To this substituents containing a chiral center in the *R*-configuration are added. Changing the substitution pattern enables to control the intramolecular interaction between the substituents. An overview of the compounds considered in this work is provided in Fig. 2. In compounds **1a** and **2a**, the sidechains and their conformational properties can be expected to be largely independent from each other. For example, steric hindrance is limited thanks to the large distance between the sidechains. Vibrational mode coupling between the sidechain vibrations may still impact the vibrational frequencies and corresponding VCD intensities though. By changing the substitution pattern we impact the conformational freedom through specific intramolecular interactions. Hence, we may introduce steric interactions between the side chains when going from **1a** to **1b** and hydrogen bonding in going from **2a** to **2b**. Differences in the performance of the ML procedure can then be attributed to the interactions introduced. The influence of a wider variety in the functional groups present in the side chains, yielding more feature-rich spectra, is examined using compounds **3** and **4**. The absence of a C_2_ axis in addition reveals the impact of the associated symmetry operation.

The obtained excellent quality of the spectral prediction suggests that ML can link the geometry of a conformation to its VCD spectrum. As such, ML can strongly reduce the effort spent in quantum chemically obtaining all VCD spectra provided the ML step has a much lower computational cost. This is indeed shown to be the case. On the other hand, unfortunately, the ML models are not transferable, not even within the same molecule but with different AC.

ML as well as DFT-based prediction of VCD spectra are quite technical fields and every step needs to be very well thought of. Because of the highly technical nature, the precise methodology including all error checks and balances used are given in the methods section and supplementary material. The main lines of the approach taken are:

- Generate minimum energy conformations using a force field for all compounds in Fig. 2 with chosen AC equal to RRRR
- Compute DFT geometries and VCD spectra using the B3PW91 functional and 6-31G(d) basis set
- Establish a training, validation, and test set per compound to train an ML model to extract from solely a conformational geometry the VCD spectrum and test hypothesis 1 (see above)
- Repeat this for all conformations of a molecule in the chosen AC and establish the time gained by using ML (hypothesis 2)
- Test the ML model for a different AC of the same molecule or even a different molecule in the same AC or different (hypothesis 3)

Admittedly, in this study, the entire usual approach involving elaborate DFT calculations is also still performed to serve as a comparison basis but the end goal is to strongly reduce the number of these calculations although some will always remain required to train the model.

## Results and discussion

For each conformer of each compound in a single, chosen AC, the VCD spectrum is computed using DFT. This basis of spectra is then used in finding the ML model as described in detail in the methods section. For each compound, the ML method is trained to allow the prediction of conformer VCD spectra solely from the geometry of the conformers. In this section, emphasis is placed on the actual results which are discussed in terms of the extent to which they (dis)prove the hypotheses formulated above.

### Hypothesis 1: machine learning can predict conformer spectra solely from molecular geometry

**Fig. 3:**
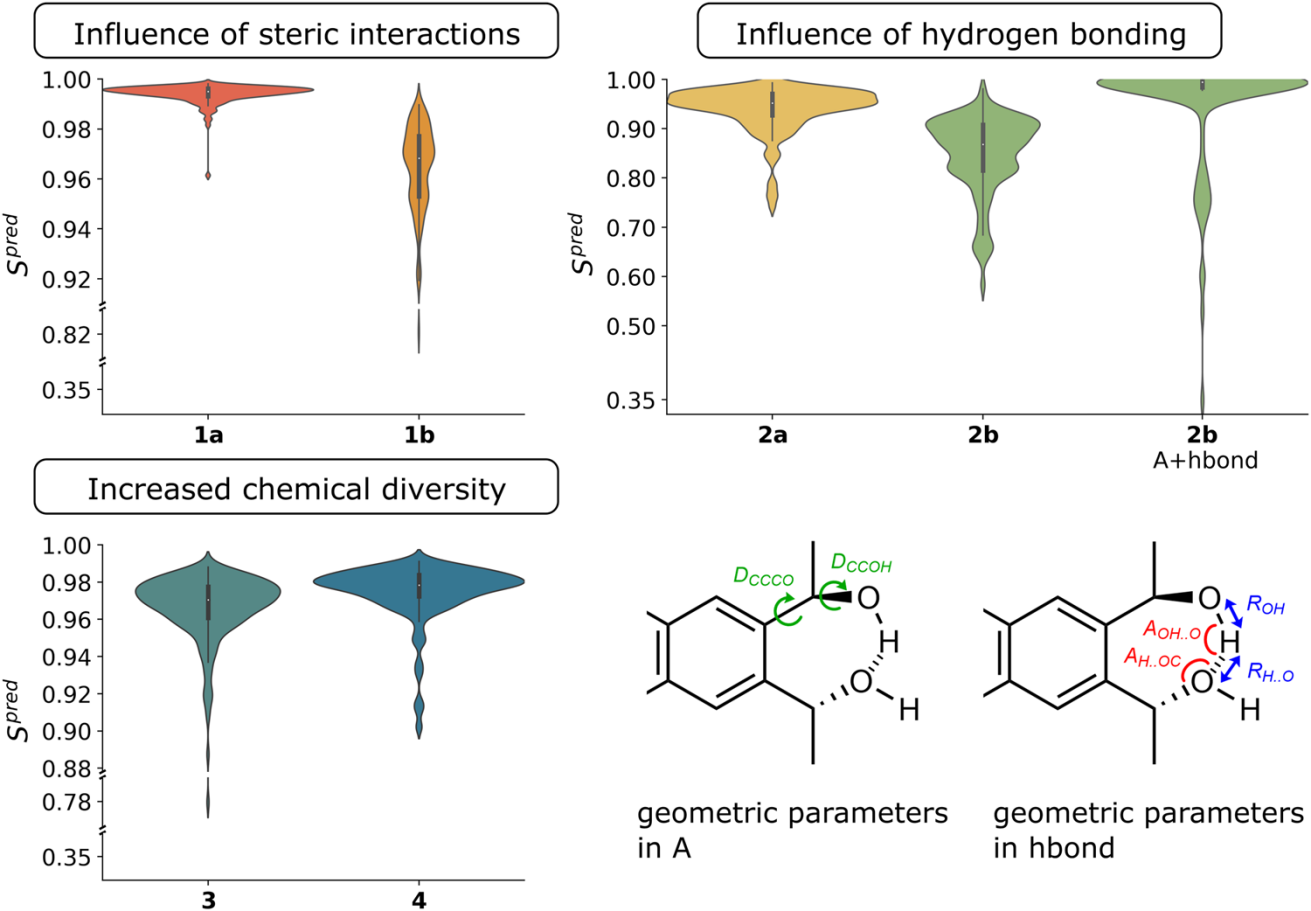
Machine learning performance. For each conformer of each compound, the similarity between the ML-predicted spectrum and the DFT computed spectrum in the test set is shown. Separate plots are used per class of compounds in Fig. 2.

The first hypothesis is that ML can learn from a dataset of conformer geometries and their VCD spectra the intricate link between both. To this end, for every compound, a training set of conformers and spectra is established so that an ML model can be obtained. This does -admittedly- mean that it is impossible to completely bypass all DFT calculations but the aim is to be able to limit the number to just enough to train a proper model. For all compounds in Fig. 2, an ML model was trained using different ratios of training, test and validation set, and the hypothesis is examined by looking at the similarity between a DFT predicted conformer spectrum and one obtained using the trained ML model. The results are presented in Fig. 3. For all applications, the molecular geometry is represented using only the sidechain dihedral angles.

As a similarity measure we use the cosine similarity measure *S*^*pred*^ which is the normalized overlap between the ML predicted and DFT computed spectrum (see Supplementary Methods 1 and 2 for details on the similarity measures). If it equals 1, the spectra are exactly the same. It can turn negative, meaning that the ML-predicted spectrum would rather agree with the enantiomer of the DFT computed spectrum. This would be detrimental for the use of ML in VCD-based AC assignment and it is gratifying that no conformers appear with negative similarities. Figure 3 are so-called violin plots. The width of the blob at every value of *S*^*pred*^ reflects how many conformers are binned within a small interval around that value. How wider the blob the more conformers have an *S*^*pred*^ in that bin.

Clearly, the procedure works very well in case of compound **1a**. The far majority of conformers comes with values around 0.99 and only a very small tail descends towards circa 0.96. To put this in perspective, the loss in exact similarity is of the order of or even better than the variation in spectrum if one compared DFT spectra for the same conformer obtained using a different basis set or functional. This shows that the ML procedure works very well. Compound **1b** is a structural isomer and there the results are somewhat less impressive. A vertically more spread out blob is obtained but the far majority of points still has an impressive similarity above 0.9. Two sets of conformers appear and one might be tempted to interpret this in terms of one collection of conformers with stronger steric hindrance and one with less, but no such connection is found (see Supplementary Discussion 2 and Supplementary Figs. 4 and 5). Compound **2a** again shows that the majority of conformers exhibits very good agreement between the ML predicted and actual DFT computed spectrum although the similarities do go down to roughly 0.75. This is still more than sufficient in the context of AC determination^56^. One could suggest that conformers with higher energy lay lower in similarity, but this is not the case (see Supplementary Discussion 3 and Supplementary Figs. 6–11). For compound **2b**, two plots are shown. The first is the result using ML training with only the sidechain dihedral angles as input. Hydrogen bonding is not well represented in this encapsulation of molecular geometry. When additional parameters are included (denoted as hbond, see Fig. 3), the violin plot shifts massively to higher similarities (see Supplementary Discussion 4 and Supplementary Figs. 12 and 13). This means that sufficient attention must be paid to what is a proper representation of a conformer geometry. Compounds **3** and **4** introduce a wider range of substituents and it is clear that the agreement between DFT and ML predicted spectra is very good.

These results reveal that ML does allow to partially replace DFT calculations. Still, for many conformations the VCD spectrum needs to be calculated using DFT as one needs a training set for each compound but once an ML model is available, the spectra of all conformations for which no DFT calculation of the VCD spectrum was performed can be computed from the ML model. A detailed study of what fraction of conformers is required for DFT VCD calculations is given in Supplementary Discussions 5 and 6, Supplementary Figs. 14 and 15, and Supplementary Table 1.

### Hypothesis 2: machine learning can significantly reduce the computational cost for AC assignment

From a practical perspective, the scientifically already valuable results above, suggest that one could significantly reduce the effort to assign an AC to an experimental sample. In practice, assigning the AC of an experimental sample requires elaborate DFT calculations for all conformers in each possible AC, composing a Boltzmann averaged VCD spectrum and comparing it to an experimental measurement. Even if for the moment, we assume that a separate ML model needs to be trained for every assumed AC, there may be a significant time gain due to the use of ML. The most time consuming part in the usual approach lies in computing the VCD spectra, much less in the geometry optimization so for now we take for granted that the geometries and Boltzmann weights are DFT computed. One could envision to also skip the step of geometry optimization and use only geometries and energies from a force field calculation but this is subject of future work. At this exploratory stage, it is important not to reach too far in ambitions to avoid conclusions could be based on partial error cancellation.

Table 1 shows the total time cost for all compounds to compute a Boltzmann weighted VCD spectrum using the classical approach and using one where part of the DFT VCD calculations are replaced by the ML-based prediction. To allow for a fair comparison, the time spent to train the ML model is also reported. The data in Table 1 is obtained using a very large fraction of DFT conformer VCD spectra (DFT spectra computed for 80% of all conformers). As the ML training step can be done quite efficiently, the relative time savings are mostly limited by the time spent in computing DFT spectra to generate the ML model. Nonetheless, significant computer time is already being saved compared to the classical approach.

**Table 1:** Computational cost reduction due to machine learning.

	DFT cost	DFT cost ML-	Cost generation	Time savings ML-
Compound	Classical approach	Aided approach	ML model	Aided approach
**1a**	7140 h	5707 h	7 h	1426 h
**1b**	5507 h	4406 h	4 h	1097 h
**2a**	2691 h	2153 h	6 h	532 h
**2b**	2025 h	1619 h	5 h	401 h
**3**	7293 h	5828 h	11 h	1454 h
**4**	4771 h	3811 h	6 h	954 h

Comparison of the cost for the Boltzmann weighted spectrum with the classical approach (using the DFT computed spectra for all conformers) and the ML-aided approach where 80% of all conformer spectra come from DFT calculations and the remaining 20% are predicted with the ML model. Cost is reported in cpu time for a Intel Xeon E5-2680v4 processor.

**Fig. 4:**
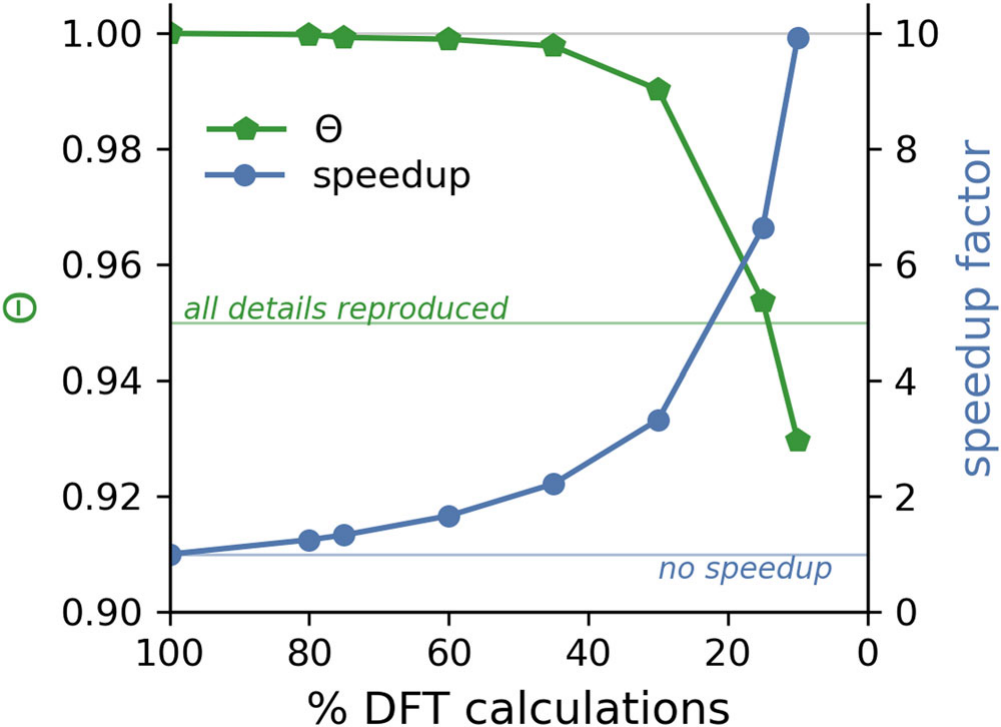
Efficiency gain due to machine learning. The relative speedup with the ML-aided approach (blue) using different percentages of DFT conformer spectra is shown for compound **4**. The similarity of the Boltzmann weighted spectrum obtained with the ML-aided approach and the one composed with all DFT conformer spectra (green) is determined for each percentage of DFT conformer spectra using the cosine similarity Θ (see Supplementary Methods 1 for details).

Computing DFT spectra for 80% of all conformers of course limits the possible time gain with ML. Hence, we also investigate the additional time savings possible if the ML model is generated using a smaller percentage of DFT computed spectra. Using fewer DFT spectra may adversely affect the similarity between the Boltzmann weighted spectrum composed with the DFT spectra of all conformers and one based on a combination of DFT spectra and ML predictions. Figure 4 shows the relative speedup for the Boltzmann weighted spectrum as a function of the percentage of DFT computed spectra, along with the similarity of the Boltzmann weighted spectrum and the one based on the DFT spectra of all conformers, for compound **4**. It is found that one can strongly reduce the percentage of DFT computed spectra without significantly affecting the resulting spectrum in the sense that the similarity to the spectrum composed with all DFT conformer spectra remains very high. At a similarity of 0.95, all details of the spectrum are still reproduced. With roughly 15% of the conformer spectra computed with DFT and used to generate the ML model, the ML-aided approach allows to retain a similarity above the threshold of 0.95 while providing a speedup with a factor of 6.6.

Similar speedup values as reported for compound **4** are also found for the other model compounds. The Boltzmann weighted spectra obtained for each compound with this approach, along with the associated speedup and similarity, are given in Supplementary Discussions 7–8 and Supplementary Figures 16–18.

### Hypothesis 3: machine learning can generate transferable models

**Fig. 5:**
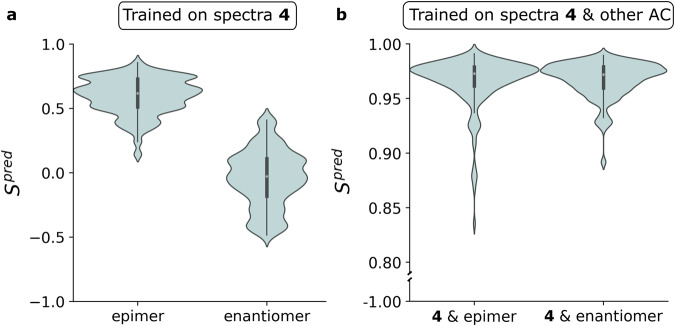
Transferability of machine learning models. **a** similarity of the predictions for the epimer and enantiomer with the ML model trained on only compound **4**. **b** similarity of the predictions from the ML model trained on a combination of the conformers of compound **4** and the conformers of either the epimer or enantiomer.

All of the above is based on individually training an ML model for a specific AC of a specific molecule. The gratifying time savings reported above could be very strongly boosted if learning an ML model for a single AC would lead to a model that can also be used for a different stereoisomer. To test this we took compound **4** where ML works excellently for a single AC (see hypothesis 1 and Fig. 3). We then switched the AC of compound **4** to both an epimer and the enantiomer, and used the ML model generated for compound **4** to predict conformer spectra for both. The results are presented in Fig. 5a. The predictions for the new stereoisomers unfortunately do not resemble the DFT conformer spectra. The ML model is far from transferable to other AC’s, especially if the conformer spectra differ significantly from the original AC. In an attempt to remedy this, one could suggest to train for multiple stereoisomers in one run. With this approach the conformer spectra of each stereoisomer are obtained with the same accuracy as for a single AC (Fig. 5b). With the current approach, the ML model can only faithfully reproduce spectra for stereoisomers it has been trained on.

**Fig. 6:**
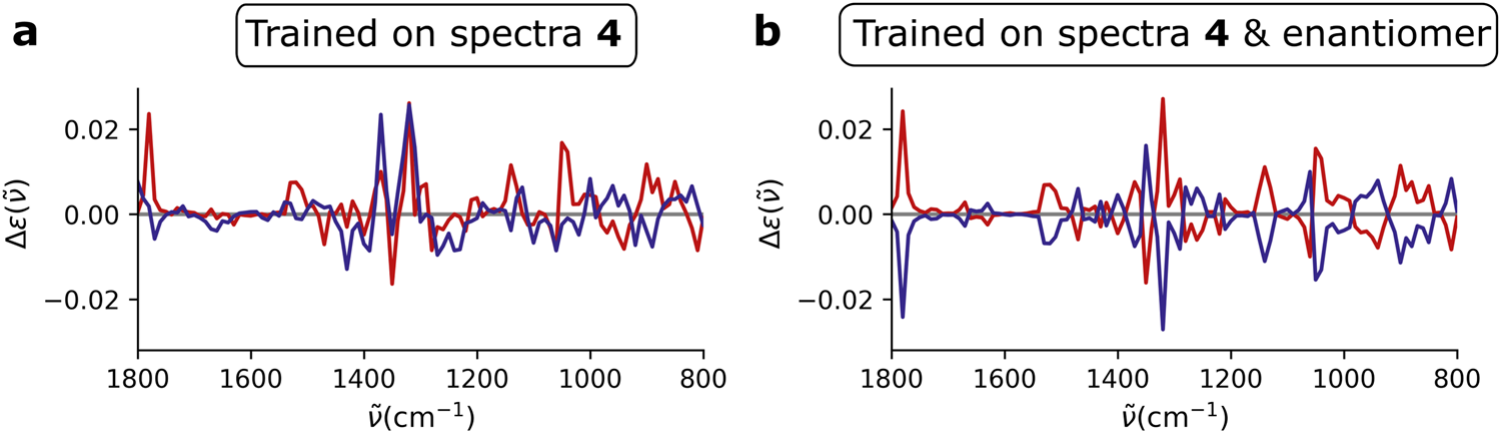
Machine learning and enantiomer spectra. **a** prediction for a selected conformer of the test set of **4** (red) and its mirror image (blue) when the ML model is trained on conformers of **4**. **b** prediction for the conformer and its mirror image when the ML model is trained on conformers of both **4** and the enantiomer.

Figure 5 also reveals a particular feature. DFT spectra are always only computed for one enantiomer of the set of enantiomers as spectra of enantiomers are mirror images. It is clear that this was not picked up when training on only one of both enantiomers. When training on both enantiomers, the question is whether enough information was sourced such that for the two mirror images of the same conformation also a mirror image spectrum is obtained from the ML model. This is indeed the case as is shown in Fig. 6 where in panel a the spectra of an enantiomeric pair of conformers is compared when only one AC was used in training. In Fig. 6b, the result is shown when both AC are included in training.

## Conclusions

The potential of ML in VCD spectroscopy to (partially) replace DFT calculations was examined. Three hypotheses have been put forward, leading to the following conclusions:

- Hypothesis 1: machine learning can predict conformer spectra solely from molecular geometries. The similarity between the DFT computed spectrum of a conformer and the spectrum predicted with ML from its geometry is very high. ML can indeed learn the intricate and hidden connection between a conformer geometry and its VCD spectrum. Though, it is up to the user to make sure that the representation of the geometry in a practical form encapsulates all the necessary input to cover intramolecular interactions.
- Hypothesis 2: machine learning can significantly reduce the computational cost of AC assignment. The present results show that the ML training step may be done quite efficiently and as a result significant time savings are possible. Obviously, it remains up to the user to determine whether the time savings compensate for the learning curve associated with proper training in ML methods.
- Hypothesis 3: machine learning can generate transferable models. The current design architecture does not result in transferable ML models, neither between molecules nor among different AC’s of the same molecule.

The current ML approach already satisfies 2 out of 3 hypotheses. Clearly, more development on the ML methodology is still needed to satisfy hypothesis 3. Nonetheless, ML shows promise as a tool for extracting the link between conformations and VCD spectra.

## Methods

### Conformational analysis and VCD DFT calculations

VCD spectra are very conformation dependent and so a molecular VCD spectrum for a chosen AC is composed of a set of conformer VCD spectra each weighted with their Boltzmann weight. Hence, to compute a proper VCD spectrum that takes into account all conformers and their Boltzmann weights, it is necessary to thoroughly sample the conformer ensemble within the chosen AC. The conformer geometries and VCD spectra also constitute the input for an ML model. In order to provide the model with an as diverse input as possible both low-energy and a substantial number of higher energy conformers are generated using a force-field-based conformer generation algorithm. The geometry of the conformers is then optimized further using DFT and VCD spectra are calculated for each conformer. The details of each step are provided below.

- Conformer generation: A set of conformers is generated using the GMMX routine^57^, which implements a stochastic search mechanism. Conformational energies are computed with the MMFF94^58^ force field as implemented in PcModel10^59^. During the stochastic search, a cut-off on the energy of the conformers equal to 40 kcal mol^−1^ is used. In practice, the generated conformers spread over a smaller range of force field energies. The high cut-off does not mean that we expect the high energy conformers to significantly impact the Boltzmann averaged spectrum but it may add to the diversity of the input for the ML stage. Second, experience shows that some interactions are not well handled at the force field stage and conformer energies may change significantly when moving to the DFT level.
- Geometry optimization and VCD spectrum generation: For each conformer, the geometry is optimized further and the VCD line spectrum is computed with the B3PW91^60^ functional, the 6-31G(d) basis set and assuming the rigid rotor, ideal gas, and harmonic approximation. These calculations are performed using Gaussian16^61^.
- Spectrum broadening and representation: The computed conformer spectra are broadened using a Lorentzian band shape with a full width at half maximum (FWHM) of 10 cm^−1^. The spectra were represented as vectors containing the molar absorbance difference ${{\Delta }}\epsilon (\tilde{\nu })={\epsilon }_{L}(\tilde{\nu })-{\epsilon }_{R}(\tilde{\nu })$ for wavenumbers $\tilde{\nu }$ ranging from 800 cm^−1^ to 1800 cm^−1^ using a sampling interval equal to the FWHM (10 cm^−1^), so a 101-dimensional vector.

The distribution of the conformer DFT energies is discussed in Supplementary Methods 3 and Supplementary Fig. 20.

### ML model architecture, training, and optimization

**Fig. 7:**
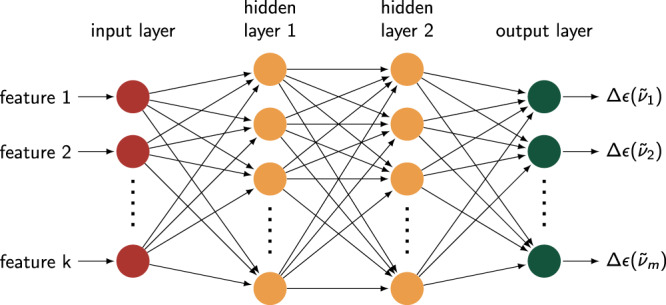
General structure of FNN. Illustration of an FNN with two hidden layers. Molecular features such as dihedral angles are provided to the input neurons (red) and VCD intensities emerge from the output neurons (green).

A fully connected Feedforward Neural Network (FNN) is used in this work to extract the link between conformer geometries and their corresponding VCD spectra. The input is a vector containing molecular features describing the geometry of the conformer (for full description see section ‘Molecular representation’) and the output is the 101-dimensional vector representing its VCD spectrum. Layers of artificial neurons, so-called hidden layers, are inserted between the input and output layer. During training, the connections between the neurons establish the link between the VCD spectrum and the conformer geometry. An illustration of an FNN with two hidden layers is shown in Fig. 7. Training a single FNN to predict VCD intensities for multiple $\tilde{\nu }$ simultaneously, improves the generalizability^62,63^ of the connections between the layers. The set of conformers for a specific AC of a single molecule is split randomly into three sets: a training, validation and test set. As mentioned earlier, the connections between the neurons are extracted from the training set. The validation set is used to optimize the so-called hyperparameters of the FNN such as its size and the algorithm used for training. The test set provides a final test of how well the FNN can predict the spectra of new conformers. Initially a default 80%:10%:10% (training:validation:test) split is used. Results of the ML approach for different splits are reported in Supplementary Discussion 5.

More technical details of the model are:

- Hyperparameter Optimization: for every application of the model the hyperparameters are optimized using Bayesian optimization. Here, a tree-structured parzen estimator optimizes the hyperparameters within the search space shown in Table 2^64^. By reoptimizing the model for every application (such as compound, representation, or training set size) we prevent data leaking from previous applications to the current model. The Bayesian optimization was implemented with Hyperopt 0.2.5^65^.

**Table 2:** Search space for FNN optimization.

Hyperparameter	Values
Number of hidden layers	$\left\{1,2,3,...,8\right\}$
Number of neurons per hidden layer	$\left\{50,60,70,...,500\right\}$
Dropout rate	$\left\{0,0.05,0.10,0.15,0.20\right\}$
Activation function	$\left\{tanh,elu,relu,selu\right\}$
Optimizer	{*adam*, *nadam*, *rmsprop*, *nesterov momentum*}
Learning rate	$\left[1{0}^{-5},1{0}^{-2}\right]$
Use of batch normalization	$\left\{True,False\right\}$
Regularization type	$\left\{{Lasso\ L}_{1},{Ridge\ L}_{2}\right\}$
Regularization strength	$\left[1{0}^{-9},1{0}^{-4}\right]$
Early stopping patience	5 epochs

Hyperparameter space considered for Bayesian optimization of the FNN. For a more detailed description of the individual concepts we refer to the documentation of Tensorflow^68^.
- Dropout/Batch Normalization: during the Bayesian optimization the tree-structured parzen estimator can choose to introduce Dropout^66^ for the hidden layers or batch normalization^67^ layers to reduce overfitting.
- Loss function: the model is trained with the mean squared error as loss function. The exact implementation of the metric is explained in Supplementary Methods 1-2.

All models are built and trained on a Xeon E5-2680v4 processor using Tensorflow 2.2.0^68^.

### Molecular representation

**Fig. 8:**
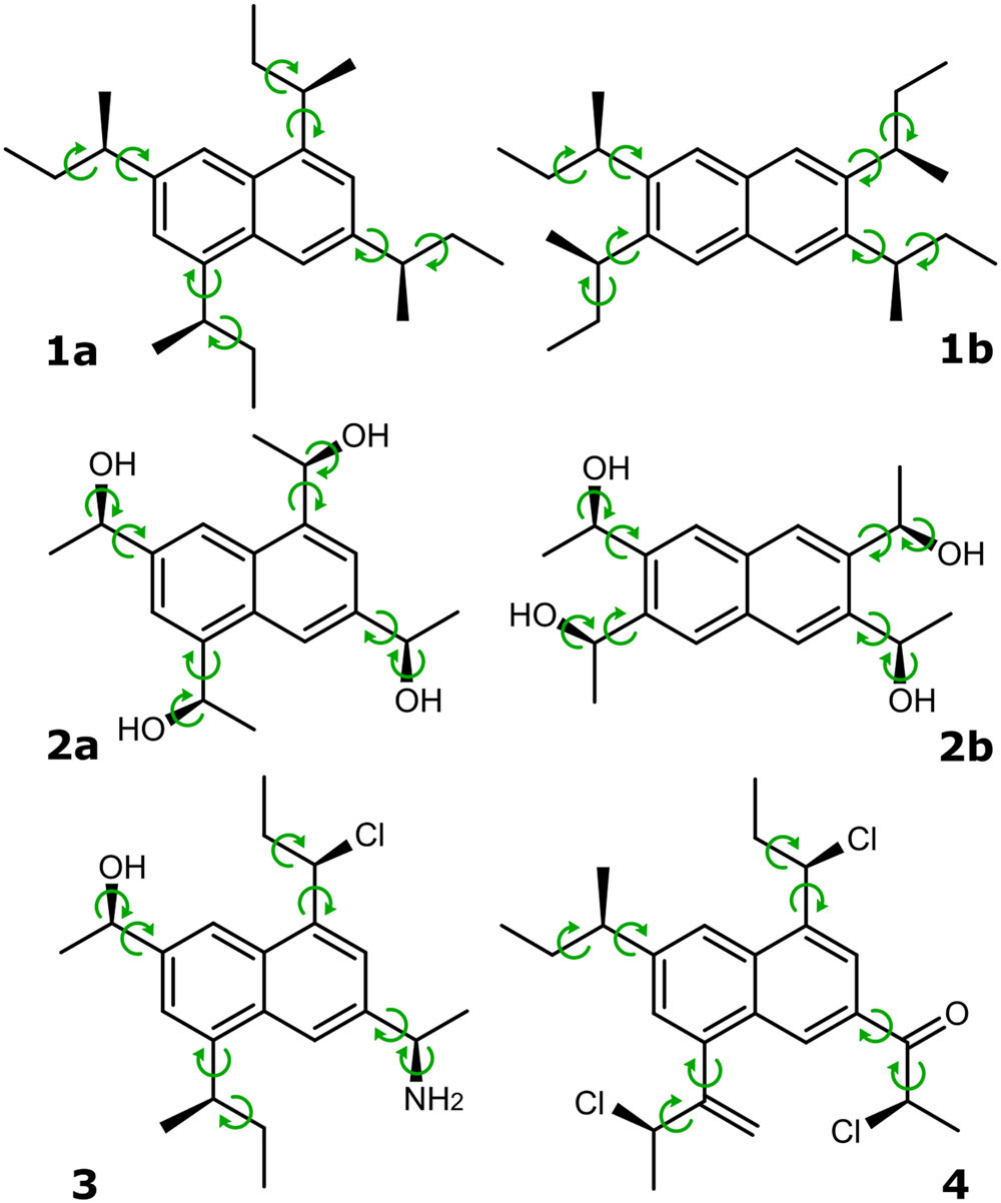
Dihedral angles representing the molecular geometry. The set of dihedral angles used to describe the conformer geometries for each compound (representation A). The orientation of the NH_2_ group of compound **3** was encoded as the bisector between the dihedral angles C-C-N(H_*B*_)-H_*A*_ and C-C-N(H_*A*_)-H_*B*_ to remove the influence of the numeric labels assigned to H_*A*_ and H_*B*_.

The ML model is trained to predict the VCD conformer spectra from the conformer geometries of each molecule in turn and for a chosen AC. Ideally, a minimal set of intramolecular coordinates that fully describes the conformation is chosen as input for the model. For each of the six compounds, the major differences between conformers of the same compound lie in the geometrical arrangement of the sidechains. Therefore, the conformer geometry is presented to the ML model as the set of dihedral angles in the sidechains shown in Fig. 8. Throughout this work we will refer to this set of dihedral angles as representation A. We expect this representation to capture most of the conformational flexibility. If the model cannot fully capture the link between conformer and spectrum with representation A, other geometric parameters are added to the representation and their influence is discussed.

Conformers of compounds **1a**/**1b**/**2a**/**2b** lacking a *C*_2_ axis can arise in two different ways by rotating the sidechains internally, resulting in degenerate conformations which share the same VCD spectrum but are presented to the ML model as different conformations with this representation. Hence, we will teach the model that the predicted spectra need to be the same for both by explicitly including both members of such pairs.

## Supplementary information

Supplementary Material

## Data Availability

The conformer dataset that supports the findings of this study is openly available at 10.5281/zenodo.8009874. The technical approach and choices are described in full detail in the supplementary material.
