# Supplementary material for: Impact of conformation and intramolecular interactions on vibrational circular dichroism spectra identified with machine learning
Source: Commun Chem. 2023 Jul 12;6:148. doi: 10.1038/s42004-023-00944-z (PMC10338531; doi:10.1038/s42004-023-00944-z)
Supplement: Supplementary file 1 — Supplementary Material [file 42004_2023_944_MOESM1_ESM.pdf]

Supporting Information

Impact of conformation and intramolecular interactions on vibrational circular dichroism spectra identified with machine learning.†

Tom Vermeyen,[*,a,b] Ana Cunha,[a] Patrick Bultinck[*,b] and Wouter Herrebout[a]

[a] *Department of Chemistry, University of Antwerp, Groenenborgerlaan 171, B-2020 Antwerpen, Belgium.*
[b] *Department of Chemistry, Ghent University, Krijgslaan 281, B-9000 Gent, Belgium.*

E-mail: patrick.bultinck@ugent.be, tom.vermeyen@uantwerpen.be

Contents

Supplementary Discussions **3**
1 S^{conf} distribution of IR & VCD spectra 3
2 Impact of steric interactions on S^{pred} for 1b 6
3 Comparison of conformer $\Delta H^0_{298.15}$ and S^{pred} 7
4 Influence of representation on performance for individual conformers of 2b . 10
5 $\overline{S^{pred}}$ on VCD conformer spectra with different data splits 12
6 Training, validation and test set MSE for different data splits 14
7 Construction of Boltzmann weighted spectrum with ML predictions 16
8 Relative speedup for Boltzmann weighted spectrum 19

Supplementary Methods **20**
1 Spectral similarity and model performance 20
2 Data scaling and influence on MSE 23
3 Energy distribution of conformers 24

Supplementary References **25**

Supplementary Discussions

1 S^{conf} distribution of IR & VCD spectra

Prior to deploying an ML model to predict conformer spectra, we need to establish the variability of spectra between conformers of the same compound. If the conformer spectra within the same AC of a single compound are all very similar, high S^{pred} values can be obtained without the ML model extracting a meaningful pattern between geometry and spectrum. If $\overline{S^{pred}}$ outclasses the mean similarity between DFT conformer spectra themselves, the model has successfully established a link between the geometry of a conformer and its spectrum. For each compound, the cosine similarity between all unique pairs of conformer spectra (S^{conf}; see equation 3 in Supplementary Methods 1) is determined. This analysis is performed for both the IR and VCD conformer spectra separately. Note that for the IR spectra S^{conf} can have values between 0 and 1 while for VCD spectra S^{conf} can have values between -1 and 1. The distribution of S^{conf} is depicted in Supplementary Figure 1 for the IR conformer spectra and Supplementary Figure 2 for the VCD conformer spectra. A violin plot is used to describe the distribution of S^{conf} for each compound. The width of the "blob" reflects the number of conformer pairs found within a small interval around that S^{conf} value and the added boxplot shows the median value and interquartile range.

The variability between IR conformer spectra is rather weak: For compound **1a** and **1b**, $\overline{S^{conf}}$ exceeds 0.9 and for compound **2a** $\overline{S^{conf}}$ is larger than 0.8. For these compounds any differences between the conformer IR spectra are subtle at best. For compounds **2b**, **3** and **4** lower $\overline{S^{conf}}$ values are obtained, though the overall sensitivity of the IR spectra to conformational differences remains limited.

The VCD conformer spectra are more sensitive to conformational differences than the IR conformer spectra. For each compound, the $\overline{S^{conf}}$ obtained for the VCD spectra is significantly lower than the $\overline{S^{conf}}$ for the corresponding IR spectra. For the VCD spectra, the largest $\overline{S^{conf}}$ is obtained for **1a** with a mean value of 0.556. Introduction of steric interactions increases the variability between conformer VCD spectra resulting in a lower $\overline{S^{conf}}$ of 0.418 (**1b**). When hydrogen bonding interactions are introduced, $\overline{S^{conf}}$ drops slightly from 0.182 (**2a**) to 0.170 (**2b**). The largest variability between conformer spectra is observed for the compounds with increased chemical diversity with $\overline{S^{conf}}$ values of 0.097 (**3**) and 0.150 (**4**). Interestingly, the lowest $\overline{S^{conf}}$ is obtained for **3** which has the largest number of possible conformers (Supplementary Figure 3).

Altogether, the VCD spectra are clearly sensitive to conformational differences. The IR spectra are less sensitive to these conformational differences which limits the added value of the ML approach compared to VCD. The focus in this paper will therefore lie on assessing the ML approach for VCD.

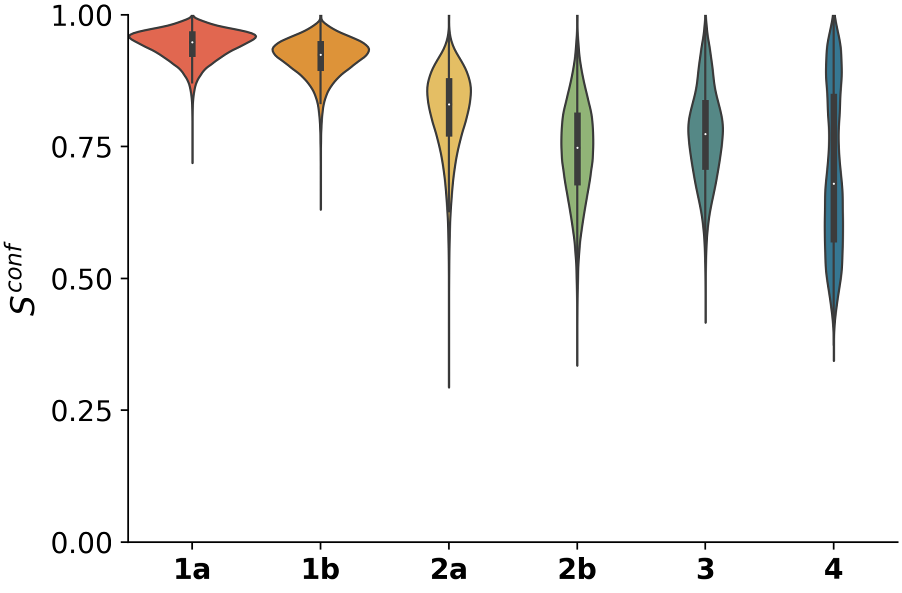

Supplementary Figure 1: Similarity of conformer IR spectra. The cosine similarity between IR spectra corresponding to unique conformer pairs are reported for each compound. For the different compounds following $\overline{S^{conf}}$ values are obtained: 0.942 (**1a**), 0.919 (**1b**), 0.818 (**2a**), 0.744 (**2b**), 0.773 (**3**), 0.703 (**4**).

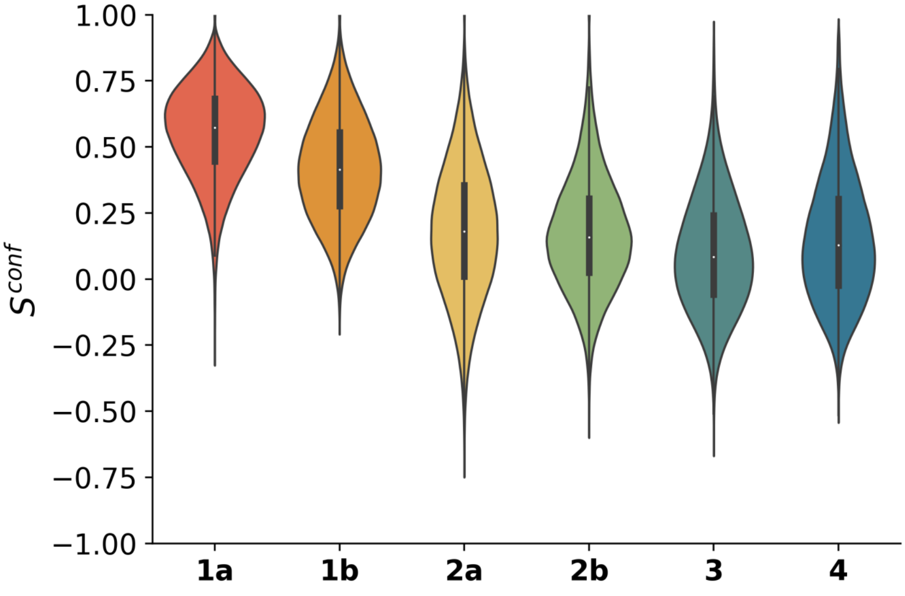

Supplementary Figure 2: Similarity of conformer VCD spectra. The cosine similarity between VCD spectra corresponding to unique conformer pairs are reported for each compound. For the different compounds following $\overline{S^{conf}}$ values are obtained: 0.556 (**1a**), 0.418 (**1b**), 0.182 (**2a**), 0.170 (**2b**), 0.097 (**3**), 0.150 (**4**).

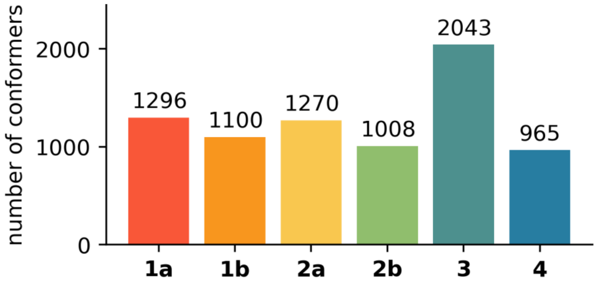

Supplementary Figure 3: Number of conformer spectra for each compound.

2 Impact of steric interactions on S^{pred} for 1b

The steric interactions between adjacent *sec*-butyl side chains for compound **1b** decreases the $\overline{S^{pred}}$ compared to compound **1a**. One could, naively, expect that this results from lower S^{pred} values for conformers with larger steric hindrance. We test this notion using the lowest H..H distance between the CH_2 and the $(CH_2)CH_3$ groups of adjacent sidechains within each individual conformer (see Supplementary Figure 4) as a metric for steric interaction. Supplementary Figure 5 shows that the S^{pred} value obtained for a conformer is not linked to the steric hindrance within said conformer.

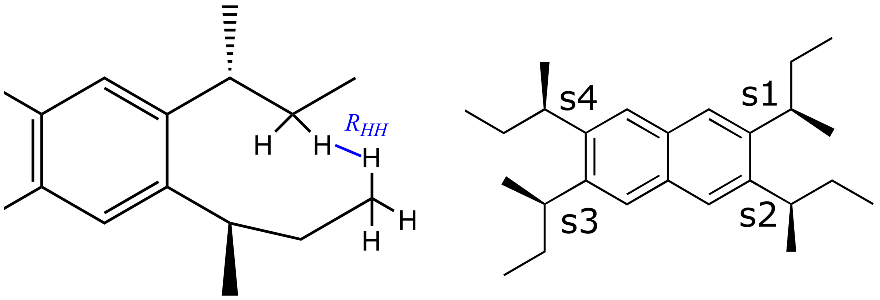

Supplementary Figure 4: Minimum H..H distance used to describe steric interactions between the sidechains. The H..H distance shown in the left panel is calculated for each pair of adjacent sidechains (s1-s2, s2-s1, s3-s4, s4-s3; see right panel) and the minimum value of these H..H distances is determined for each individual conformer.

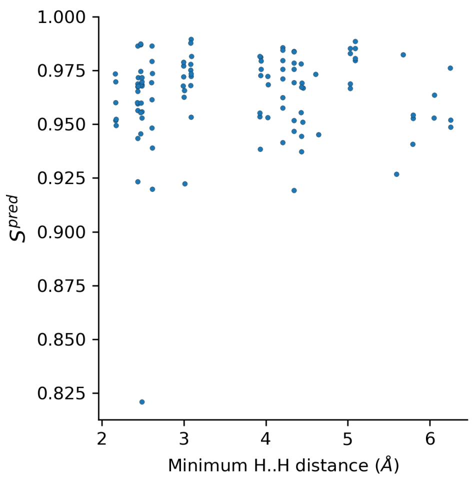

Supplementary Figure 5: Influence of minimum H..H distance on S^{pred} for each conformer in the test set of **1b**.

3 Comparison of conformer $\Delta H^0_{298.15}$ and S^{pred}

In Supplementary Figures 6-11 $\Delta H^0_{298.15}$ and S^{pred} are compared for every test set conformer of a compound. Figure dimensions were adapted for Supplementary Figure 7 and 9 to accommodate for the larger range of $\Delta H^0_{298.15}$ for **1b** and of S^{pred} for **2b**. In general, there is no pattern showing that lower S^{pred} values are obtained for conformers with larger $\Delta H^0_{298.15}$. Thus, the accuracy of the ML model predictions is not biased towards conformers with lower $\Delta H^0_{298.15}$.

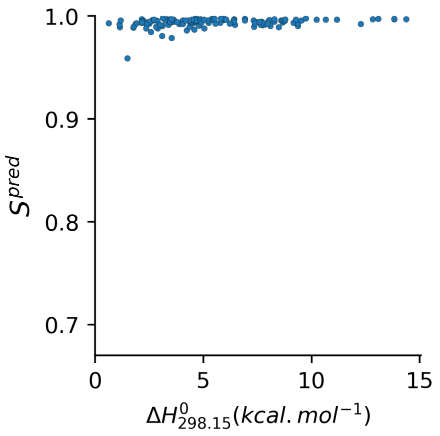

Supplementary Figure 6: Comparison of $\Delta H^0_{298.15}$ and S^{pred} for the test set of compound **1a**.

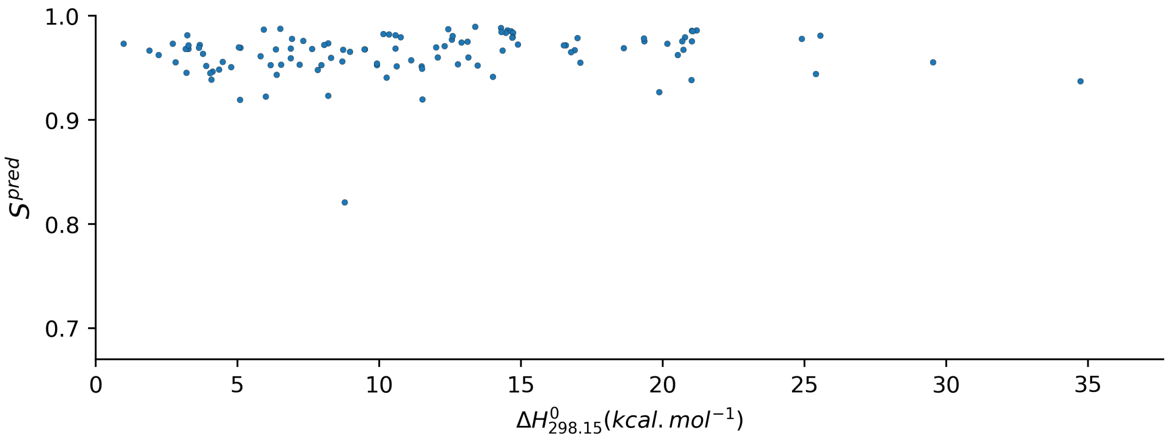

Supplementary Figure 7: Comparison of $\Delta H^0_{298.15}$ and S^{pred} for the test set of compound **1b**.

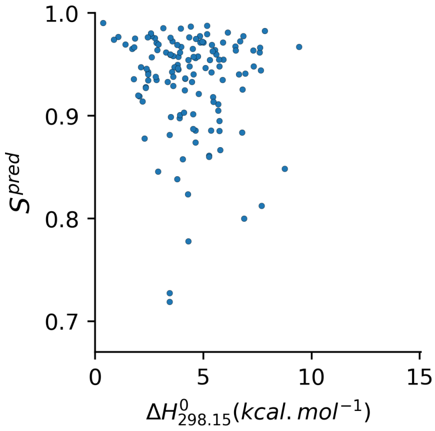

Supplementary Figure 8: Comparison of $\Delta H^0_{298.15}$ and S^{pred} for the test set of compound **2a**.

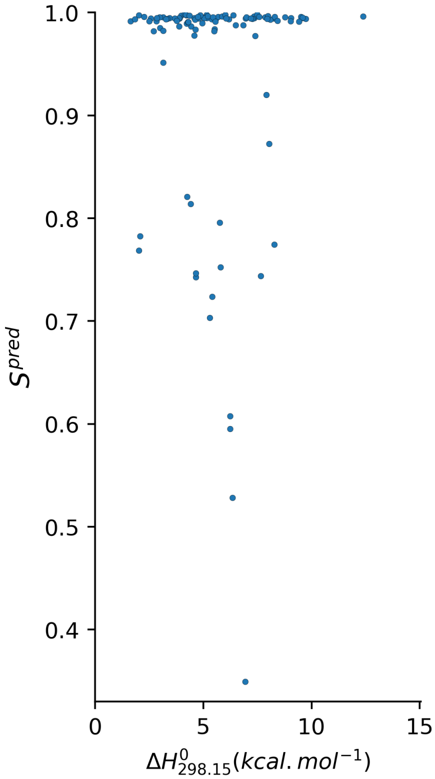

Supplementary Figure 9: Comparison of $\Delta H^0_{298.15}$ and S^{pred} for the test set of compound **2b**.

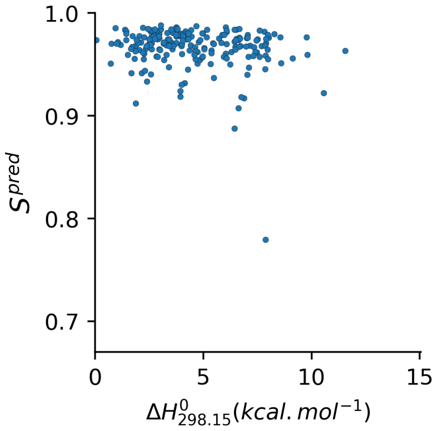

Supplementary Figure 10: Comparison of $\Delta H^0_{298.15}$ and S^{pred} for the test set of compound **3**.

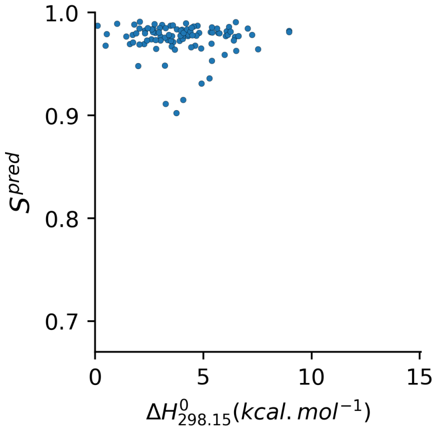

Supplementary Figure 11: Comparison of $\Delta H^0_{298.15}$ and S^{pred} for the test set of compound **4**.

4 Influence of representation on performance for individual conformers of 2b

For compound **2b** lower S^{pred} values are obtained with representation A compared to the other compounds. Representation A might not sufficiently capture the hydrogen bonding in a way understandable to the ML model. To improve the accuracy of the ML predicted spectra, the additional parameters shown in figure 3 are used to describe the geometry of the conformers. Doing so, the S^{pred} shifts to higher values as a result (see Supplementary Figure 12). For a large majority of the conformers in the test set S^{pred} increases with the additional parameters (see Supplementary Figure 13), though for a handful of conformers lower S^{pred} values are obtained with the new representation. Including these hbond parameters does not negatively impact generalization as indicated by the generalization factor reported in Supplementary Discussion 6. To summarize: including the hydrogen bond angles and distances allows the ML model to extract a more detailed link between the conformer and its VCD spectrum though a small fraction of the conformers do not fit in the pattern obtained with this new representation.

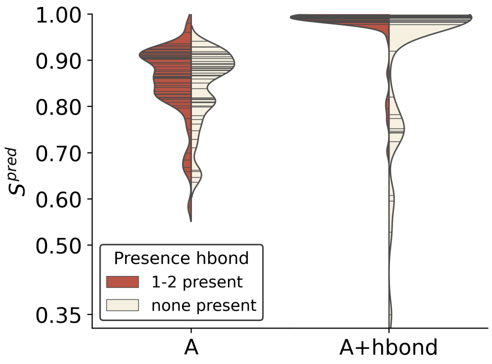

Supplementary Figure 12: Distribution of the S^{pred} values for conformers in the test set of compound **2b** with (red) and without (white) intramolecular hydrogen bonds for both representations. Individual S^{pred} values are shown as horizontal bars within the violin plots.

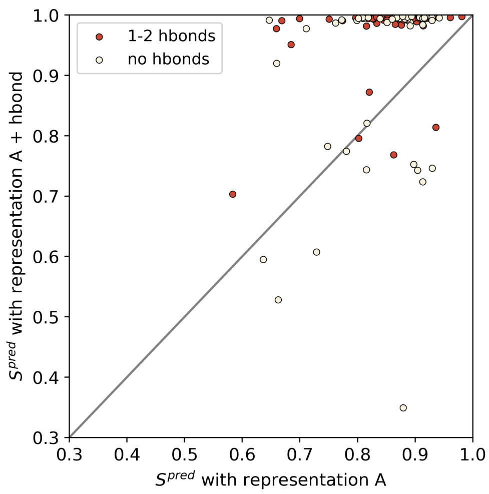

Supplementary Figure 13: S^{pred} values obtained with representation A and A+hbond for each conformer in the test set of compound **2b**. Presence of an intramolecular hydrogen bond within a conformer is denoted by color. Conformers lying above the diagonal have increased S^{pred} values upon adding the hbond parameters.

5 $\overline{S^{pred}}$ on VCD conformer spectra with different data splits

As demonstrated in the section covering hypothesis 1 (see figure 3), excellent performance can be obtained for the different compounds with the default data split of 80:10:10 (training:validation:test). The ML model can clearly establish the link between conformer and spectrum. As the cost of the entire ML workflow is equal to computing 1-3 DFT spectra for this data split, the test set spectra are obtained at a fraction of the computational cost of the conventional DFT procedure. With a large majority of the conformers used as training samples, the relative speedup for obtaining all DFT conformer spectra for a single compound remains rather limited. To establish the relative speed-up obtainable whilst maintaining good predictive quality, the performance of the ML approach is examined for different data splits in this section.

The training and optimization procedure used in the previous sections, is repeated for all compounds while incrementally relocating conformers from the training set to the validation set. Repeating the optimization of the ML model ensures that no data leakage occurs (i.e. all information from the original training set is erased) and allows to decrease model complexity to accommodate for smaller training sets. The same test set is used across the different splits. Doing so, $\overline{S^{pred}}$ is established in a consistent manner and the influence of the training set size is isolated. Representation A+hbond is used for compound **2b** and representation A for the other compounds. The $\overline{S^{pred}}$ values obtained for the different splits and compounds are shown in Supplementary Figure 14.

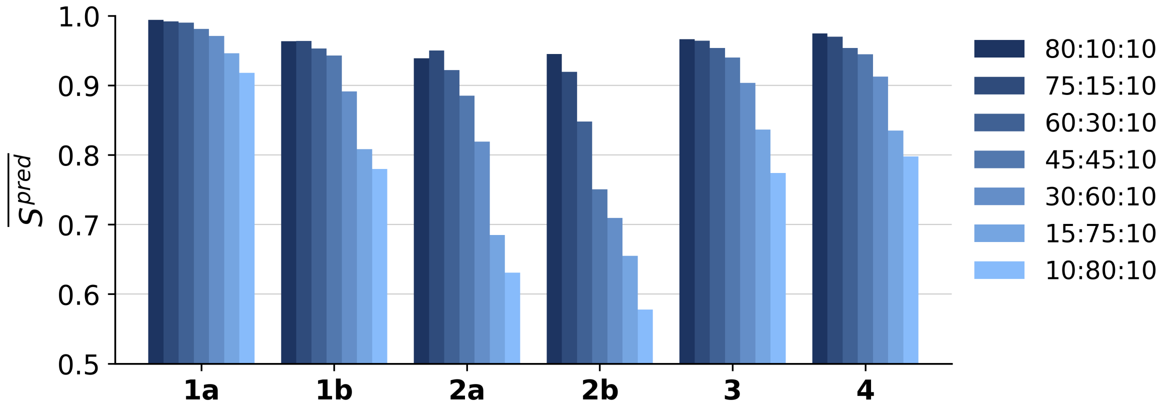

Supplementary Figure 14: Performance of the ML approach for different training set sizes. For each compound and data split, $\overline{S^{pred}}$ is shown. Training set size is denoted by color.

For **1a** excellent $\overline{S^{pred}}$ is retained across the different splits. Even when only 10% of the conformers is used for training, the test set spectra are reproduced with a $\overline{S^{pred}}$ of 0.918. For **1b** the conformer spectra are predicted with excellent $\overline{S^{pred}}$ when at least 45% of the conformers reside in the training set. For **2a** and, to a larger extent, **2b** $\overline{S^{pred}}$ drops quickly when less training samples are provided. While a training set size of 60% still yields excellent $\overline{S^{pred}}$ for **2a**, with the same training set size a $\overline{S^{pred}}$ of 0.848 is obtained for **2b**. For **3** and **4**, the decrease in $\overline{S^{pred}}$ with smaller training sets is less steep: both compounds retain excellent $\overline{S^{pred}}$ if at least 30% of the spectra are included in the training set. These $\overline{S^{pred}}$ values are

very impressive as for **3** and **4** the conformer spectra varied the most (see Supplementary Discussion 1).

The influence of vibrational mode delocalization provides an explanation for the steeper performance drop for **2a** compared to **3** and **4**. Whereas the majority of the vibrational modes within the considered wavenumber range involve only a single sidechain (along with the naphthalene moiety) for **3** and **4**, for **2a** the vibrational modes are delocalized over the entire compound. As discussed in the main paper, the sidechains are largely independent from each other in conformer space. However, they do correlate through the delocalized vibrational modes, along with the corresponding vibrational frequencies and VCD intensities. Providing a large majority of the spectra for training, this complex pattern can still be extracted by the ML model. With a small training set, too few examples are provided to the ML model to properly learn these correlations. While the same degree of delocalization is expected for **1a**, the conformer VCD spectra are more similar (see Supplementary Discussion 1). Thus, obtaining good performance with a smaller training set is relatively less challenging.

The influence of intramolecular interactions is more pronounced for smaller training sets. Correlation between sidechains in conformer space complicates the spectral prediction problem. In case of hydrogen bonding $\overline{S^{pred}}$ drops more sharply. As intramolecular hydrogen bonding has a strong influence on the vibrational modes, this correlation is even stronger and more complex in **2b**. Thus, when less examples of these correlations are provided, properly accounting for the different correlations becomes more challenging for the ML model.

6 Training, validation and test set MSE for different data splits

In this section the MSE metrics are reported for the training (MSE_{train}), validation (MSE_{val}) and test set (MSE_{test}). While the performance on the test set is the ultimate test of the ML approach, the ratios between the MSE metrics (see equation 1 and 2) can also be of interest for future reference. The use of these ratios was inspired by the work of Röbel[1,2]. The ratios are shown in Supplementary Figure 15 and the values of the MSE metrics are reported in Supplementary Table 1.

$$\rho_{train} = \frac{MSE_{test}}{MSE_{train}} \tag{1}$$

$$\rho_{val} = \frac{MSE_{test}}{MSE_{val}} \tag{2}$$

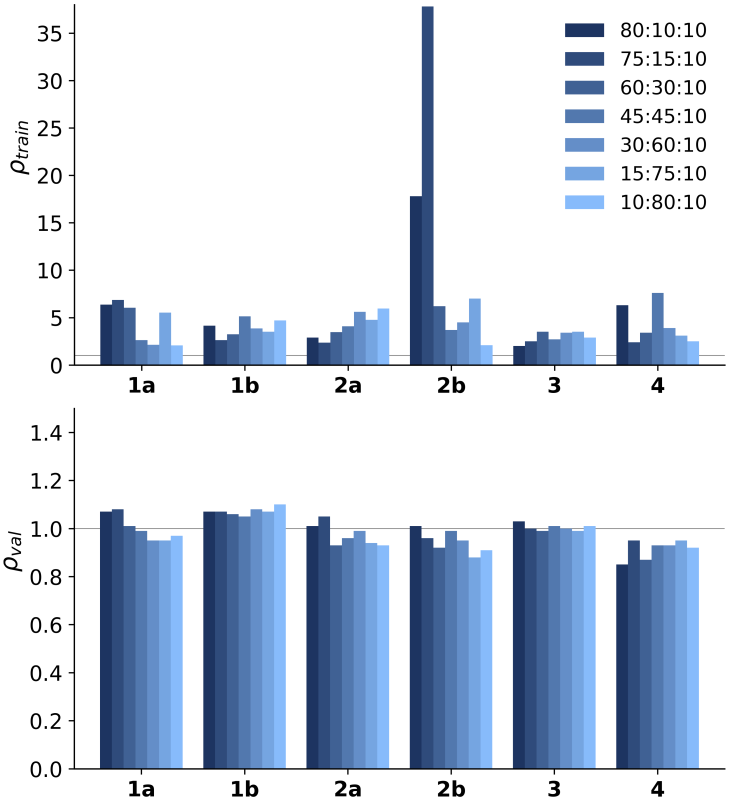

Supplementary Figure 15: Bar plot of ρ_{train} (top) and ρ_{val} (bottom) for each compound and training set size (denoted by color). Representation A+hbond was used for compound **2b** and representation A for the other compounds.

compound	representation	training set size (%)	ρ_{train}	ρ_{val}	MSE_{train}	MSE_{val}	MSE_{test}	$\overline{S^{pred}}$
**1a**	A	80	6.4	1.07	0.002	0.011	0.012	0.994
**1a**	A	75	6.9	1.08	0.002	0.015	0.016	0.992
**1a**	A	60	6.0	1.01	0.003	0.020	0.020	0.990
**1a**	A	45	2.6	0.99	0.014	0.038	0.037	0.981
**1a**	A	30	2.1	0.95	0.027	0.060	0.057	0.971
**1a**	A	15	5.5	0.95	0.019	0.113	0.107	0.946
**1a**	A	10	2.1	0.97	0.077	0.164	0.160	0.918
**1b**	A	80	4.1	1.07	0.017	0.065	0.070	0.964
**1b**	A	75	2.6	1.07	0.027	0.067	0.072	0.964
**1b**	A	60	3.2	1.06	0.029	0.088	0.093	0.953
**1b**	A	45	5.1	1.05	0.021	0.103	0.108	0.943
**1b**	A	30	3.9	1.08	0.054	0.194	0.210	0.891
**1b**	A	15	3.5	1.07	0.104	0.339	0.364	0.808
**1b**	A	10	4.7	1.10	0.086	0.368	0.405	0.780
**2a**	A	80	2.9	1.01	0.033	0.095	0.096	0.939
**2a**	A	75	2.4	1.05	0.036	0.081	0.085	0.950
**2a**	A	60	3.5	0.93	0.036	0.134	0.125	0.922
**2a**	A	45	4.1	0.96	0.047	0.201	0.192	0.885
**2a**	A	30	5.6	0.99	0.054	0.307	0.303	0.819
**2a**	A	15	4.8	0.94	0.103	0.521	0.490	0.685
**2a**	A	10	6.0	0.93	0.094	0.599	0.559	0.631
**2b**	A+hbond	80	17.8	1.01	0.006	0.106	0.107	0.945
**2b**	A+hbond	75	37.8	0.96	0.004	0.158	0.151	0.919
**2b**	A+hbond	60	6.2	0.92	0.042	0.282	0.260	0.848
**2b**	A+hbond	45	3.7	0.99	0.112	0.419	0.413	0.751
**2b**	A+hbond	30	4.5	0.95	0.106	0.499	0.472	0.709
**2b**	A+hbond	15	7.0	0.88	0.076	0.605	0.534	0.655
**2b**	A+hbond	10	2.1	0.91	0.302	0.701	0.635	0.578
**3**	A	80	2.0	1.03	0.032	0.065	0.067	0.966
**3**	A	75	2.5	1.00	0.029	0.070	0.070	0.964
**3**	A	60	3.5	0.99	0.026	0.091	0.090	0.954
**3**	A	45	2.7	1.01	0.043	0.113	0.114	0.940
**3**	A	30	3.4	1.00	0.054	0.182	0.182	0.904
**3**	A	15	3.5	0.99	0.086	0.302	0.300	0.836
**3**	A	10	2.9	1.01	0.136	0.389	0.393	0.774
**4**	A	80	6.3	0.85	0.007	0.056	0.048	0.975
**4**	A	75	2.4	0.95	0.024	0.059	0.057	0.970
**4**	A	60	3.4	0.87	0.025	0.098	0.084	0.953
**4**	A	45	7.6	0.93	0.014	0.111	0.103	0.945
**4**	A	30	3.9	0.93	0.041	0.172	0.159	0.913
**4**	A	15	3.1	0.95	0.093	0.304	0.288	0.835
**4**	A	10	2.5	0.92	0.139	0.379	0.347	0.798

Supplementary Table 1: Mean squared errors on the train, validation and test set (standard scaled) spectra for the different compounds and splits.

7 Construction of Boltzmann weighted spectrum with ML predictions

In the section covering hypothesis 1 we have established that VCD conformer spectra can be accurately predicted with an ML model. A remaining question is the extent to which the Boltzmann weighted spectrum obtained with the classical approach used in most applications of VCD, can be approximated using the predictions of the ML model. We address this question for each compound by constructing a Boltzmann weighted spectrum $\Delta\epsilon^{ML}(\tilde{\nu})$ that uses all DFT enthalpies along with the DFT spectra of the training set and ML predicted spectra for the validation and test sets. The Boltzmann weighted spectrum obtained with the classical approach i.e. using DFT enthalpies and DFT spectra for all conformers, is referred to as $\Delta\epsilon^{DFT}(\tilde{\nu})$. The similarity of $\Delta\epsilon^{DFT}(\tilde{\nu})$ and $\Delta\epsilon^{ML}(\tilde{\nu})$, referred to as Θ, is determined for each compound (using equation 8 in Supplementary Methods 1) and shown in Supplementary Figure 16. For the 80:10:10 split, $\Delta\epsilon^{ML}(\tilde{\nu})$ and $\Delta\epsilon^{DFT}(\tilde{\nu})$ are completely indistinguishable ($\Theta > 0.999$). So, by replacing 20% of the conformer DFT spectra with ML predictions, we introduce the significant time savings reported in Table 1 without losing even the tiniest details of $\Delta\epsilon^{DFT}(\tilde{\nu})$.

The results in Supplementary Discussion 5 indicate that with fewer conformers in the training set the accuracy of the ML predicted conformer spectra does decrease. By constructing $\Delta\epsilon^{ML}(\tilde{\nu})$ with a larger fraction of ML predicted spectra, we test whether a similar drop is observed for Θ. Note that $\Delta\epsilon^{ML}(\tilde{\nu})$ is constructed for each of the smaller training sets using the ML model obtained with said training set. Again, $\Delta\epsilon^{ML}(\tilde{\nu})$ reproduces $\Delta\epsilon^{DFT}(\tilde{\nu})$ with excellent accuracy across all different splits. When at least 30% of all conformers (or 45% for **2b**) are included in the training set, $\Delta\epsilon^{ML}(\tilde{\nu})$ replicates even the tiniest details of $\Delta\epsilon^{DFT}(\tilde{\nu})$ ($\Theta \geq 0.99$). A slight decrease in Θ is noted for even smaller training sets but Supplementary Figure 17 shows that $\Delta\epsilon^{DFT}(\tilde{\nu})$ and $\Delta\epsilon^{ML}(\tilde{\nu})$ remain very similar. So, for these compounds $\Delta\epsilon^{DFT}(\tilde{\nu})$ is approximated very well by leveraging the ML predicted conformer spectra.

Curiously, Θ decreases more steeply for **4** compared to **3** despite the larger $\overline{S^{conf}}$ and larger/similar $\overline{S^{pred}}$ for **4**. A possible explanation for this lies in the cancellation of VCD intensities during Boltzmann averaging of conformer spectra. VCD intensities of opposite sign for different conformers can partially cancel each other. Errors in the predicted VCD intensities can be averaged out in a similar manner. The degree of error compensation will depend on the sign and (Boltzmann weighted) magnitude of the errors and thus differ between compounds.

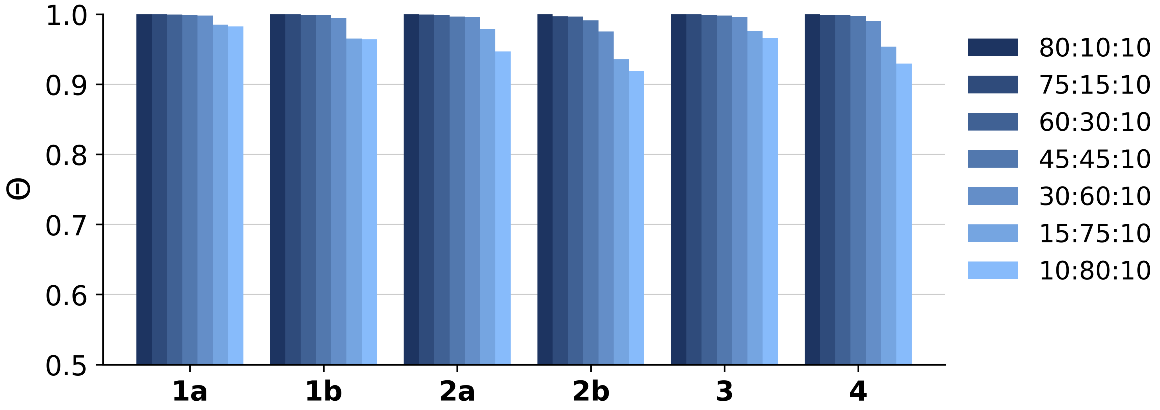

Supplementary Figure 16: Similarity Θ of the Boltzmann weighted spectra $\Delta\epsilon^{DFT}(\tilde{\nu})$ and $\Delta\epsilon^{ML}(\tilde{\nu})$ for each compound and split. Training set size is denoted by color.

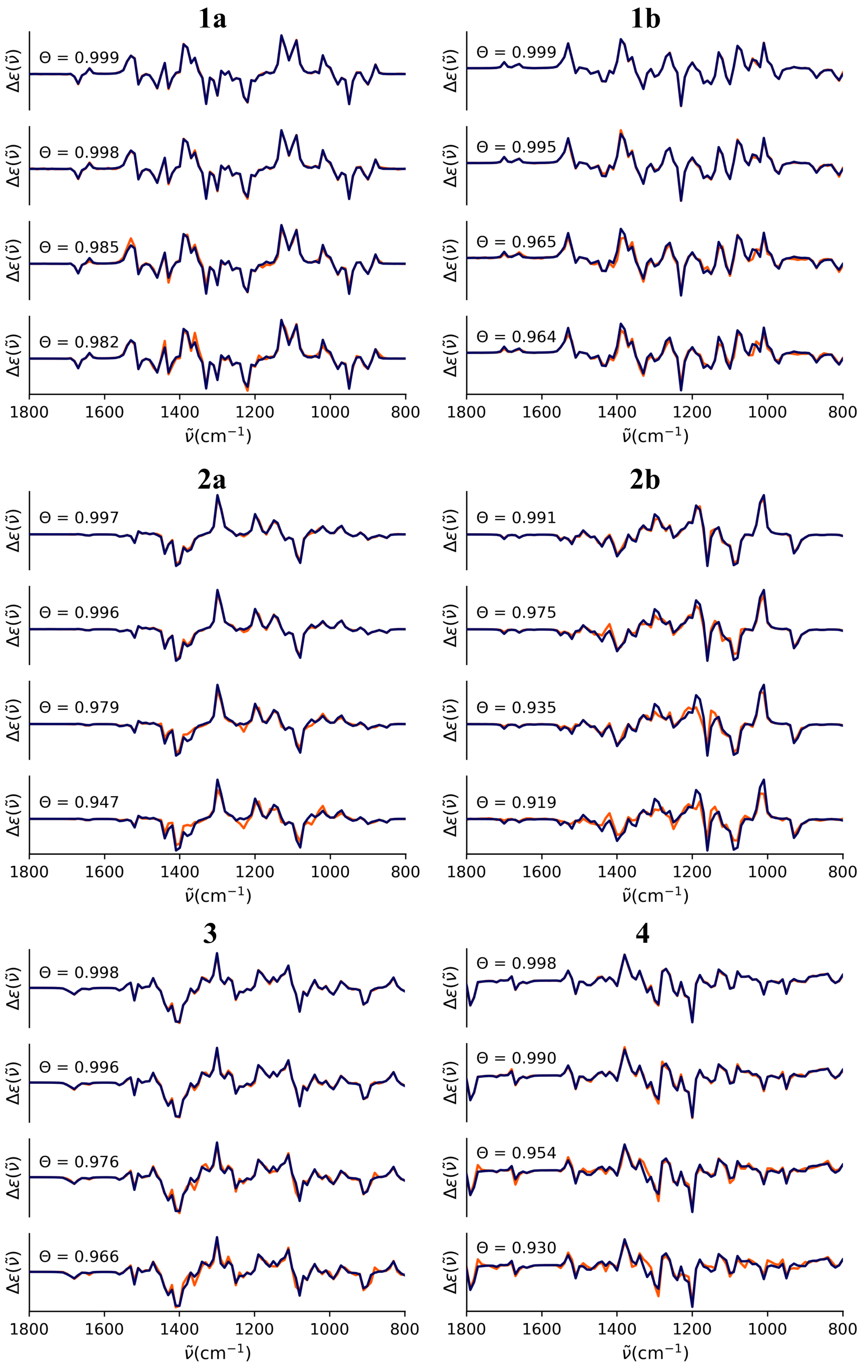

Supplementary Figure 17: Comparison of $\Delta\epsilon^{DFT}(\tilde{\nu})$ (blue) and $\Delta\epsilon^{ML}(\tilde{\nu})$ (orange) for the different compounds (as denoted) and smaller training set sizes. For each compound $\Delta\epsilon^{ML}(\tilde{\nu})$ is shown as obtained for the following training set sizes (from top to bottom): 45%, 30%, 15% and 10%. Values of Θ are reported for each split and compound in the corresponding figures.

8 Relative speedup for Boltzmann weighted spectrum

The ratio in the cost of the classical approach (using only DFT spectra) and the ML-aided approach (including time spent in the ML model generation), referred to as the relative speedup, is determined for each compound and split. The result is shown in Supplementary Figure 18 for all compounds along with the corresponding similarity Θ introduced in Supplementary Discussion 7. As the ML training step can be done very efficiently, the speedup is inversely proportional to the training set size. Very significant speedup may be obtained by limiting the percentage of conformations for which the spectrum is computed by DFT and used for training the ML model. As this percentage grows smaller, some similarity loss is found but putting the limit at 0.95, it is clear that one may strongly lower this percentage before the spectrum starts to deviate substantially from the spectrum obtained using only DFT conformer spectra (see Supplementary Figure 17).

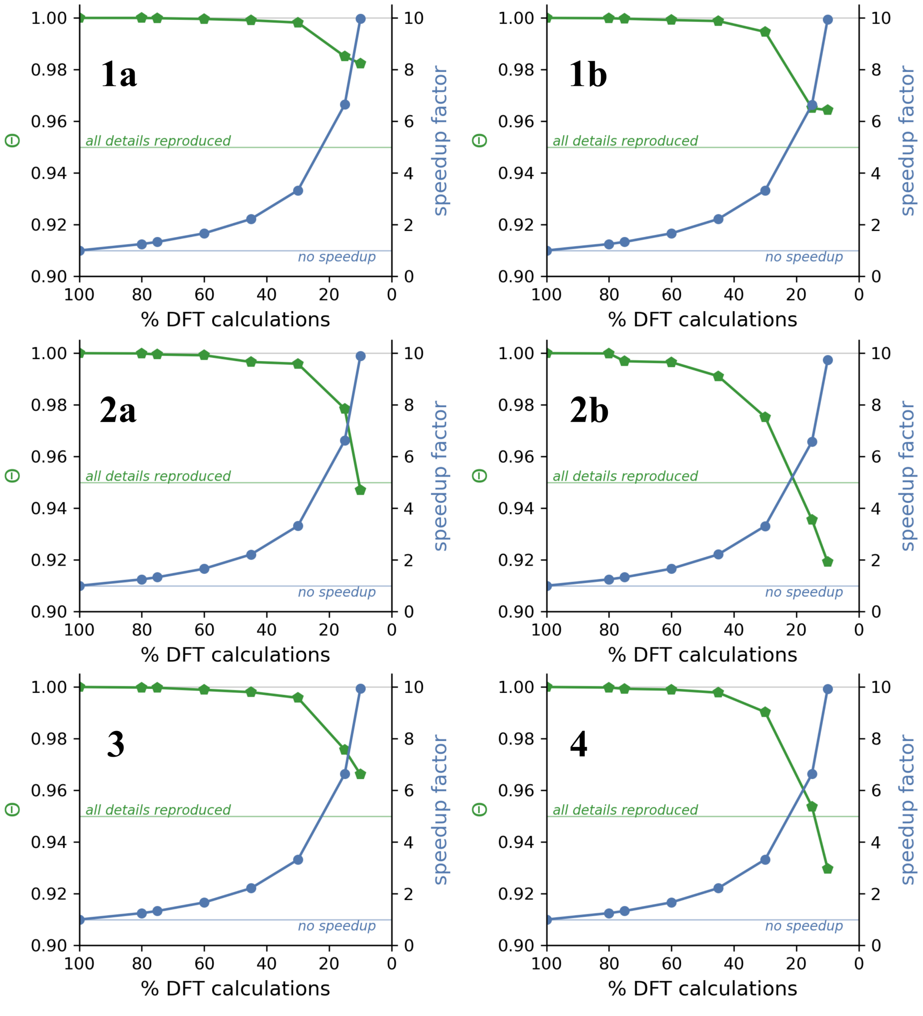

Supplementary Figure 18: Comparison of the relative speedup obtained with the ML-aided approach (blue) and Θ (green) for each compound and training set size.

Supplementary Methods

1 Spectral similarity and model performance

In this paper we probe the ability of ML to predict conformer spectra directly from the geometry of the conformers. The metrics used to express the similarity of different spectra and to train the ML model play a pivotal role and hence a more detailed discussion on these metrics is warranted. The cosine similarity (also known as the overlap integral or Carbó index) is often used within the VCD community to define the relative similarity of two spectra.[3] The similarity metric adopts values between -1 and 1, where 1 indicates the spectra to be identical and -1 identifies the spectra as perfect mirror images. The similarity metrics used within this paper are detailed below and a summary is provided in table 2 for future reference. Their use will become clear in later sections.

- A first similarity measure reflects the relative similarity of DFT calculated conformer spectra. Here, the cosine similarity S_{ij}^{conf} of two calculated spectra $\Delta\epsilon_i^{calc}(\tilde{\nu})$ and $\Delta\epsilon_j^{calc}(\tilde{\nu})$ is determined for a pair of conformers i and j (equation 3). The mean value of S^{conf} over all unique pairs of the M conformers is denoted $\overline{S^{conf}}$ (equation 4).

$$S_{ij}^{conf} = \frac{\sum_{\tilde{\nu}=800}^{1800} \left(\Delta\epsilon_i^{calc}(\tilde{\nu}) \cdot \Delta\epsilon_j^{calc}(\tilde{\nu}) \right)}{\sqrt{\sum_{\tilde{\nu}=800}^{1800} \left(\Delta\epsilon_i^{calc}(\tilde{\nu}) \right)^2} \sqrt{\sum_{\tilde{\nu}=800}^{1800} \left(\Delta\epsilon_j^{calc}(\tilde{\nu}) \right)^2}} \tag{3}$$

$$\overline{S^{conf}} = \frac{2}{M(M-1)} \sum_{i}^{M-1} \sum_{j>i}^{M} S_{ij}^{conf} \tag{4}$$

- The ML model is trained using the mean squared error (MSE) between the scaled DFT conformer spectra $\Delta\epsilon^{calc,sc}(\tilde{\nu})$ and the predictions for the scaled spectra $\Delta\epsilon^{pred,sc}(\tilde{\nu})$ (equation 5) for all N conformers in a set (training, validation or test). A detailed explanation of the scaling methodology is provided in Supplementary Methods 2. After training and optimization, the predictions for the scaled spectra are transformed to the same scale as $\Delta\epsilon^{calc}(\tilde{\nu})$, resulting in $\Delta\epsilon^{pred}(\tilde{\nu})$.

$$MSE = \frac{1}{101 \cdot N} \sum_{i}^{N} \sum_{\tilde{\nu}=800}^{1800} \left(\Delta\epsilon_i^{calc,sc}(\tilde{\nu}) - \Delta\epsilon_i^{pred,sc}(\tilde{\nu}) \right)^2 \tag{5}$$

  We do not train the ML model using a cosine similarity as it is a relative similarity metric. If trained with a cosine similarity, the ML model only learns to recreate the shape of a conformer spectrum but not the overall VCD intensity $\sum_{\tilde{\nu}=800}^{1800} (\Delta\epsilon_i^{calc}(\tilde{\nu}))^2$ for each conformer i. The mismatch in this intensity between the DFT and ML predicted spectra will be different for each conformer. As a result, the ML predictions can no longer be used to build a Boltzmann weighted spectrum.

- Once the ML model is trained and optimized, the relative similarity of a DFT calculated spectrum $\Delta\epsilon_i^{calc}(\tilde{\nu})$ of conformer i and the spectrum predicted by the ML model for said conformer $\Delta\epsilon_i^{pred}(\tilde{\nu})$ is expressed using the cosine similarity S_i^{pred} (equation 6). The mean value of S^{pred} over all N conformers within the test set is denoted $\overline{S^{pred}}$ (equation 7).

$$S_i^{pred} = \frac{\sum_{\tilde{\nu}=800}^{1800} \left(\Delta\epsilon_i^{calc}(\tilde{\nu}) \cdot \Delta\epsilon_i^{pred}(\tilde{\nu})\right)}{\sqrt{\sum_{\tilde{\nu}=800}^{1800} \left(\Delta\epsilon_i^{calc}(\tilde{\nu})\right)^2} \sqrt{\sum_{\tilde{\nu}=800}^{1800} \left(\Delta\epsilon_i^{pred}(\tilde{\nu})\right)^2}} \tag{6}$$

$$\overline{S^{pred}} = \frac{1}{N} \sum_{i}^{N} S_i^{pred} \tag{7}$$

In the context of this paper we see the ML prediction as excellent for S^{pred} values exceeding 0.9 as any remaining errors in the spectrum are hardly visible to the human eye. In Supplementary Figure 19 we show this using some examples for different S^{pred} values.

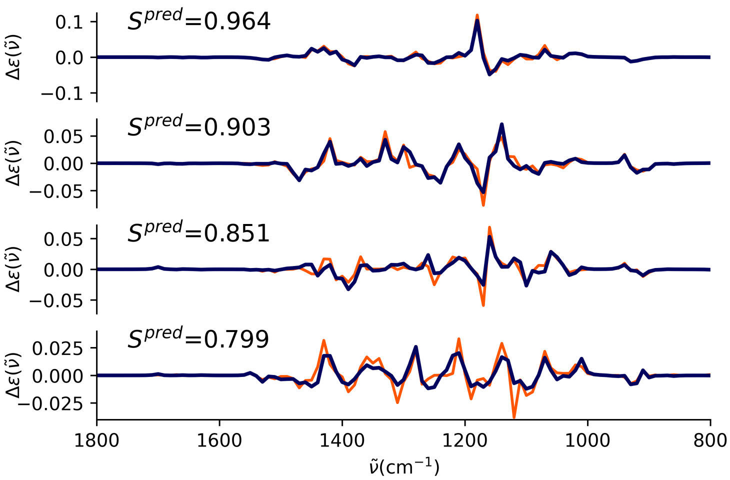

Supplementary Figure 19: Comparison of DFT conformer spectra (orange) and corresponding ML predicted spectra (blue) for different S^{pred} values. The DFT spectra and ML predictions are taken from Supplementary Discussion 4.

- Eventually, the Boltzmann weighted spectrum $\Delta\epsilon^{DFT}(\tilde{\nu})$ computed entirely from DFT spectra is compared with the Boltzmann weighted spectrum $\Delta\epsilon^{ML}(\tilde{\nu})$ where DFT spectra of the training set are combined with the ML predicted spectra for the remaining conformers. The similarity of both Boltzmann weighted spectra is determined with Θ (equation 8).

$$\Theta = \frac{\sum_{\tilde{\nu}=800}^{1800} \left(\Delta\epsilon^{DFT}(\tilde{\nu}) \cdot \Delta\epsilon^{ML}(\tilde{\nu})\right)}{\sqrt{\sum_{\tilde{\nu}=800}^{1800} \left(\Delta\epsilon^{DFT}(\tilde{\nu})\right)^2} \sqrt{\sum_{\tilde{\nu}=800}^{1800} \left(\Delta\epsilon^{ML}(\tilde{\nu})\right)^2}} \tag{8}$$

metric	description	use
S^{conf}	similarity of DFT conformer spectra for all unique conformer pairs of a single compound.	conformational sensitivity of VCD.
$\overline{S^{conf}}$	mean value of S^{conf}.	
MSE	mean squared error for the ML predicted spectra for the conformers in a set.	training and optimization of the ML model.
S^{pred}	similarity of DFT conformer spectra and corresponding ML predictions for each conformer in the test set.	describes ML model performance for new conformers.
$\overline{S^{pred}}$	mean value of S^{pred}.	
Θ	similarity of the Boltzmann weighted spectrum obtained with only DFT conformer spectra and the one obtained with DFT conformer spectra for the training set and ML predicted spectra for the validation and test set.	describes the accuracy of the Boltzmann weighted spectrum when a portion of the conformer spectra are replaced with ML predictions.

Supplementary Table 2: Overview of similarity metrics used throughout this paper.

2 Data scaling and influence on MSE

It is common practice in ML applications to standardize the features in the dataset such that each feature (e.g. a dihedral angle of a compound) has a mean value of 0 and a standard deviation of 1. This feature scaling typically improves the training of the ML model and is even required for many ML algorithms.[4–6] To prevent issues during training as a result of the low intensity of the VCD conformer spectra, we also scale the conformer spectra $\Delta\epsilon^{calc}(\tilde{\nu})$ for each compound during the training of the ML model. Using the scaling methodology detailed below we ensure that the MSE obtained for the scaled conformer spectra $\Delta\epsilon^{calc,sc}(\tilde{\nu})$ (see equation 5) remains proportional to the mean squared error if conformer spectra of original scale $\Delta\epsilon^{calc}(\tilde{\nu})$ were used instead. The scaling methodology is applied to each compound separately.

First, we determine the mean value of $\Delta\epsilon^{calc}(\tilde{\nu})$ over all M conformers of a compound for each $\tilde{\nu}$ separately and refer to it as $\mu(\tilde{\nu})$ (equation 9).

$$\mu(\tilde{\nu}) = \frac{1}{M}\sum_{i}^{M} \Delta\epsilon_i^{calc}(\tilde{\nu}) \tag{9}$$

Next, we define s as the standard deviation of $\Delta\epsilon_i^{calc}(\tilde{\nu})$ over all conformers and all $\tilde{\nu}$ (equation 10), with ω as the mean value of $\Delta\epsilon_i^{calc}(\tilde{\nu})$ over all conformers and all $\tilde{\nu}$ (equation 11).

$$s = \sqrt{\frac{1}{101 \cdot M - 1}\sum_{\tilde{\nu}=800}^{1800}\sum_{i}^{M}\left(\omega - \Delta\epsilon_i^{calc}(\tilde{\nu})\right)^2} \tag{10}$$

$$\omega = \frac{1}{101 \cdot M}\sum_{\tilde{\nu}=800}^{1800}\sum_{i}^{M} \Delta\epsilon_i^{calc}(\tilde{\nu}) \tag{11}$$

The scaled DFT spectrum for a conformer i, denoted as $\Delta\epsilon_i^{calc,sc}(\tilde{\nu})$, is obtained by subtracting $\mu(\tilde{\nu})$ from $\Delta\epsilon_i^{calc}(\tilde{\nu})$ and dividing the result by s.

$$\Delta\epsilon_i^{calc,sc}(\tilde{\nu}) = \frac{\Delta\epsilon_i^{calc}(\tilde{\nu}) - \mu(\tilde{\nu})}{s} \tag{12}$$

During training, the ML model learns to predict $\Delta\epsilon^{calc,sc}(\tilde{\nu})$ from the conformer geometries, so the predictions made by the ML model (denoted $\Delta\epsilon^{pred,sc}(\tilde{\nu})$) will be of similar scale as $\Delta\epsilon^{calc,sc}(\tilde{\nu})$. These predictions are brought back to the same scale as $\Delta\epsilon^{calc}(\tilde{\nu})$ using equation 13 and the resulting $\Delta\epsilon^{pred}(\tilde{\nu})$ are the ML predicted spectra used in equation 6 and in Supplementary Discussion 7 to construct a Boltzmann weighted spectrum.

$$\Delta\epsilon_i^{pred}(\tilde{\nu}) = s \cdot \Delta\epsilon_i^{pred,sc}(\tilde{\nu}) + \mu(\tilde{\nu}) \tag{13}$$

3 Energy distribution of conformers

As discussed in the methods section, the conformers are generated for each compound with a force field using a maximum energy window of 40 kcal mol^{-1}. This does not necessarily mean that the conformer energies actually span such a range. Additionally, the relative energies obtained for the conformers with a force field or using DFT (after geometry optimization) are likely different. As the final Boltzmann weighted spectrum discussed in later sections will be based on the DFT enthalpies, we are mainly interested in the range of DFT-based enthalpies that the conformers occupy. For each compound, the enthalpy values discussed are relative to the lowest-enthalpy conformer of said compound. The enthalpy distributions in Supplementary Figure 20 show that nearly all conformers are found within a 10 kcal mol^{-1} window for all compounds but **1b** and the distributions are centered around 5 kcal mol^{-1}. For **1b** half of the conformers are found within a 10 kcal mol^{-1} window and most of the remaining conformers lie between between 10 and 20 kcal mol^{-1}. The broader enthalpy distribution for the conformers of **1b**, compared to **1a**, can be attributed to the steric interactions between the sidechains. The influence of the steric interactions on the performance of the ML approach is discussed in Supplementary Discussion 2.

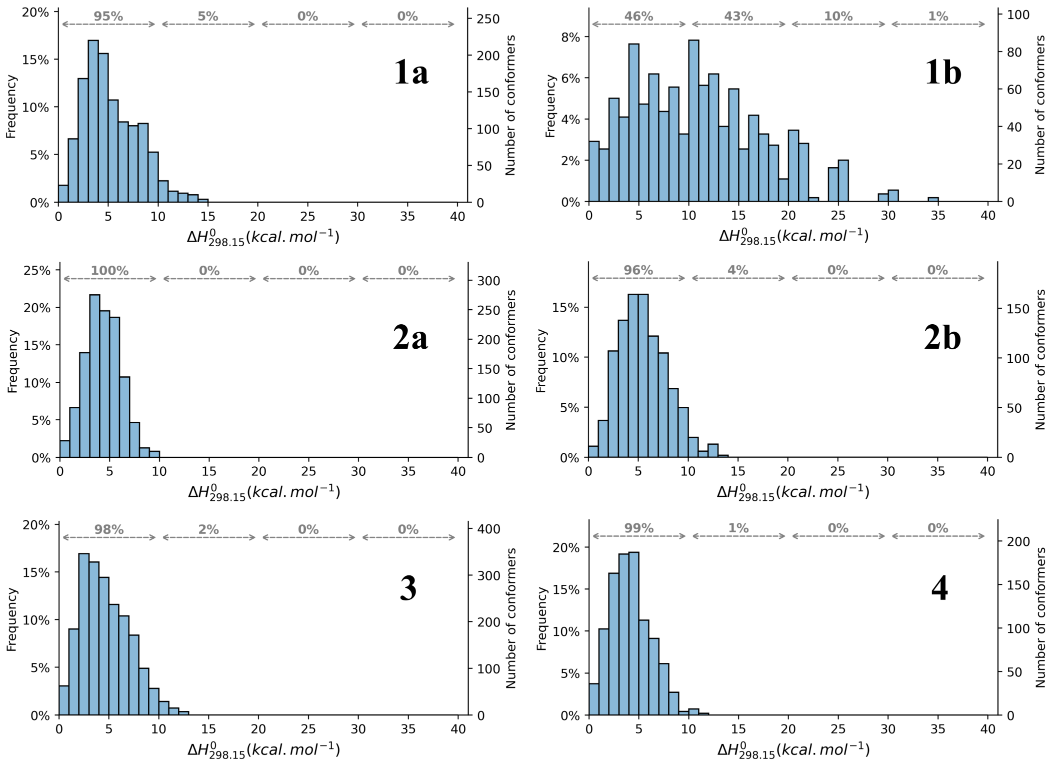

Supplementary Figure 20: Distribution of the enthalpy, relative to the lowest conformer enthalpy for each compound, for the different conformers of the same compound. For each 10 kcal mol^{-1} bin (i.e. 0-10, 10-20, 20-30 and 30-40 kcal mol^{-1}), the fraction of conformers present within this bin is reported.

Supplementary References

[1] A. Röbel, Dynamic pattern selection for faster learning and controlled generalization of neural networks, 1994.

[2] A. Engelbrecht, Computational Intelligence, John Wiley & Sons Ltd, Chichester, United Kingdom, 2nd edn., 2007, pp. 95–97.

[3] E. Debie, E. De Gussem, R. K. Dukor, W. Herrebout, L. A. Nafie and P. Bultinck, ChemPhysChem, 2011, **12**, 1542–1549.

[4] F. Pedregosa, G. Varoquaux, A. Gramfort, V. Michel, B. Thirion, O. Grisel, M. Blondel, P. Prettenhofer, R. Weiss, V. Dubourg, J. Vanderplas, A. Passos, D. Cournapeau, M. Brucher, M. Perrot and E. Duchesnay, J. Mach. Learn. Res., 2011, **12**, 2825–2830.

[5] S. Ioffe and C. Szegedy, Proceedings of the 32nd International Conference on Machine Learning, Lille, France, 2015, pp. 448–456.

[6] D. Singh and B. Singh, Appl. Soft Comput., 2020, **97**, 105524.
